# Fabricating Microfluidic Co‐Cultures of Immortalized Cell Lines Uncovers Robust Design Principles for the Simultaneous Formation of Patterned, Vascularized, and Stem Cell‐Derived Adipose Tissue

**DOI:** 10.1002/smll.202501834

**Published:** 2025-06-17

**Authors:** Ashley R. Murphy, Rose Ann Franco, Mark C. Allenby

**Affiliations:** ^1^ School of Chemical Engineering Faculty of Engineering Architecture and Information Technology The University of Queensland St Lucia 4072 Australia

**Keywords:** adipose tissue, co‐culture, gradient, microfluidics, tissue engineering, vascularization

## Abstract

In vitro co‐culture processes supporting simultaneous formation of vessel networks alongside differentiation toward mature parenchymal tissue have numerous clinical and agricultural applications but remain unrealized due to contrasting culture requirements. Of specific interest is lab‐grown vascularized adipose tissue to study diabetes, obesity, metabolic syndrome, and cardiovascular diseases, and to advance cultivated meat technologies. A microfluidic 3D hydrogel culture device capable of supporting live‐imaging of fluorescent reporter cell lines and generating counter‐current gradients of vasculogenic and adipogenic growth factors is reported. For the first time, experimental conditions capable of reproducibly forming diverse microvascular networks from telomerase immortalized endothelial and mesenchymal stem cells in both 2D and 3D hydrogel‐embedded cultures are reported. This novel microfluidic culture design demonstrates the generation of growth factor environments which support the 3D co‐formation of integrated robust microvascular networks and lipid‐producing adipocytes after 31‐days gradient culture. Microvascular networks substantially support parenchymal stromal cell differentiation to mature adipose tissue (67.4% lipid coverage), unachieved in avascular cultures (1.86% lipid coverage). It is attempted to validate the co‐culture model by applying inhibitors of vessel‐mediated lipogenesis (spermidine and VO‐OHpic), which are demonstrated to be ineffective in this novel human preclinical model.

## Graphical abstract

Murphy *et al* present an optimized co‐culture protocol for immortalized cell line vasculogenesis. Here, immortalized co‐cultures form reproducible microvessel networks across 31‐days, while gradient microchips enable simultaneous mesenchymal stem cell (MSC) adipogenesis and endothelial cell (EC) vasculogenesis. MSC differentiation to dense, lipid‐laden adipose tissue relies on EC co‐culture, and this formation of human vascularized adipose tissue is unaffected by murine regulators.

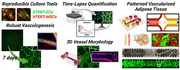

## 1 Introduction

Rapid development in miniaturized tissue and organ‐like technology represents remarkable progress toward mimicking the form and function of native biological structures and realizing the ultimate goal of restoring damaged tissue or whole organs.^[^ ^1^ ^]^ However, the limited ability of nutrients and oxygen to diffuse into dense cellular structures can often lead to cell necrosis, inhomogeneous tissue development and limited size.^[^ ^2^ ^]^ Vasculature is the human body's inherent plumbing network which supports the transport of nutrients, salts, oxygen, cells and waste. Additionally, endothelium‐derived paracrine (angiocrine) signaling, such as anti‐inflammatory and pro‐inflammatory factors during homeostasis and disease, is critical to parenchymal tissue development, maintenance and repair.^[^ ^3^ ^]^ More recently, endothelium‐secreted small molecules, metabolites and proteins have been demonstrated to regulate critical metabolic processes in parenchymal‐specific tissue such as skeletal muscle, the liver and adipose tissue.^[^ ^4^, ^5^, ^6^, ^7^ ^]^ Without functional and physiologically‐relevant vascular networks, or the means to actively incorporate host vasculature post‐implantation, the development of in vitro tissue and organ products may never progress toward clinically relevant scales and physiological complexities.^[^ ^8^ ^]^

Widespread 2D in vitro tissue culture techniques remain limited in supporting the study of pathophysiological contributions by vascular networks on parenchymal tissue.^[^ ^9^ ^]^ Endothelial cells (ECs) are typically grown in mono‐culture, requiring unique nutrients and growth factors often incompatible with the expansion or differentiation of other cell types.^[^ ^10^ ^]^ Additionally, 2D cultures deliver over saturated nutrients, growth factors and remove waste products as a batch exchange, leading to homogenous nutrient distributions with limited ability to support endothelial‐parenchymal tissue development.^[^ ^10^ ^]^ In contrast, our body's complex vascular interactions and heterogenous distributions of nutrients, growth factors, and cells evolve in a highly orchestrated manner and facilitate tissue morphogenesis during development and wound healing.^[^ ^11^ ^]^ The inability of current in vitro models to reproducibly create vascularized parenchymal tissue using basic culture protocols remains a hurdle for the translation of biomanufactured tissue.

Simple partitioned culture systems such as membrane culture inserts are often used to co‐culture two cell types, however, active cell–cell interactions and controlled establishment of 3D gradients is often not achievable. Microfluidic culture devices are increasingly used to establish, control and maintain nutrient gradients for cell and tissue growth.^[^ ^12^ ^]^ Mesenchymal stem cells (MSCs) have been shown to facilitate (EC) vasculogenesis in numerous co‐culture models and are additionally multipotent and therefore able to differentiate toward connective tissue lineages such as bone, cartilage and adipose.^[^ ^13^ ^]^ Utilising the differentiation of resident MSCs in vasculogenic co‐cultures has the potential to produce vascularised and mature connective tissues, a goal that remains unrealized due to conflicting media requirements.

White adipose tissue (WAT) is the most abundant adipose tissue in the human body, primarily comprised of 80–90% (v/v) adipocytes with the remainder attributed to vasculature containing ECs, pericytes, fibroblasts, preadipocytes, macrophages and other blood cells.^[^ ^14^, ^15^ ^]^ Traditionally, the study of adipose tissue is conducted in monolayer culture, in the absence of these supporting cell types,^[^ ^16^ ^]^ despite WAT vascular networks having critical roles in controlling adipocyte nutrient uptake, facilitating adipogenesis and regulating adipose tissue expansion.^[^ ^17^ ^]^ Recent 3D co‐cultures of adipose progenitors and microvascular cells have been developed, however, these require multi‐step differentiation processes unable to support the formation microvascular networks alongside differentiating adipose tissue.^[^ ^18^ ^]^ Simultaneous formation is critical to model adipo‐vascular cross‐talk, whose dysfunction results in conditions such as diabetes, obesity, metabolic syndrome and cardiovascular diseases,^[^ ^19^, ^20^, ^21^, ^22^, ^23^, ^24^ ^]^ which highlights the need for physiologically relevant human in vitro models of vascularized adipose tissue.

Using bespoke microfluidic device configurations, we establish spatiotemporal counter‐current gradients of vasculogenic and adipogenic culture environments across a 3D hydrogel‐supported co‐culture system. By co‐culturing highly reproducible human telomerase reverse transcriptase (hTERT) immortalized human adipose‐derived mesenchymal stem cells (hAD‐MSCs) and green fluorescent protein‐labeled hTERT immortalized human aortic endothelial cells (GFP‐hAECs), we report conditions required to form robust and optimal microvascular networks in 2D and suspended in 3D culture environments. Additionally, we utilize weeklong time‐lapse microscopy to extensively quantify and characterize vascular morphogenesis and provide informed observations regarding its mechanism. We explore the ability of gradient culture systems to support the co‐formation of microvascular networks and adipogenic differentiation of MSCs and report the enhancing effects of vascular‐mediated adipogenesis. We then apply our human preclinical vascularized adipose tissue model to identify the impact of known vasculo‐adipo regulators.

## 2 Results

### 2.1 hAECs and hAD‐MSCs Spontaneously Form Robust Microvascular Networks in Optimized 2D Culture Conditions

Immunocytochemical detection confirmed the expression of the phenotypical markers CD31 and CD90 in GFP‐hAEC and hAD‐MSC monocultures, respectively. CD31 was detected in the plasma membrane and cell nucleus of sub‐confluent GFP‐hAEC cultures (Figure S1, Supporting Information) and CD90 similarly in sub‐confluent hAD‐MSC cultures (Figure S2, Supporting Information). hAD‐MSCs demonstrated a Ki‐67 positivity of 86.27% (calculated from Figure S2B. Supporting Information).

**Figure 1:**
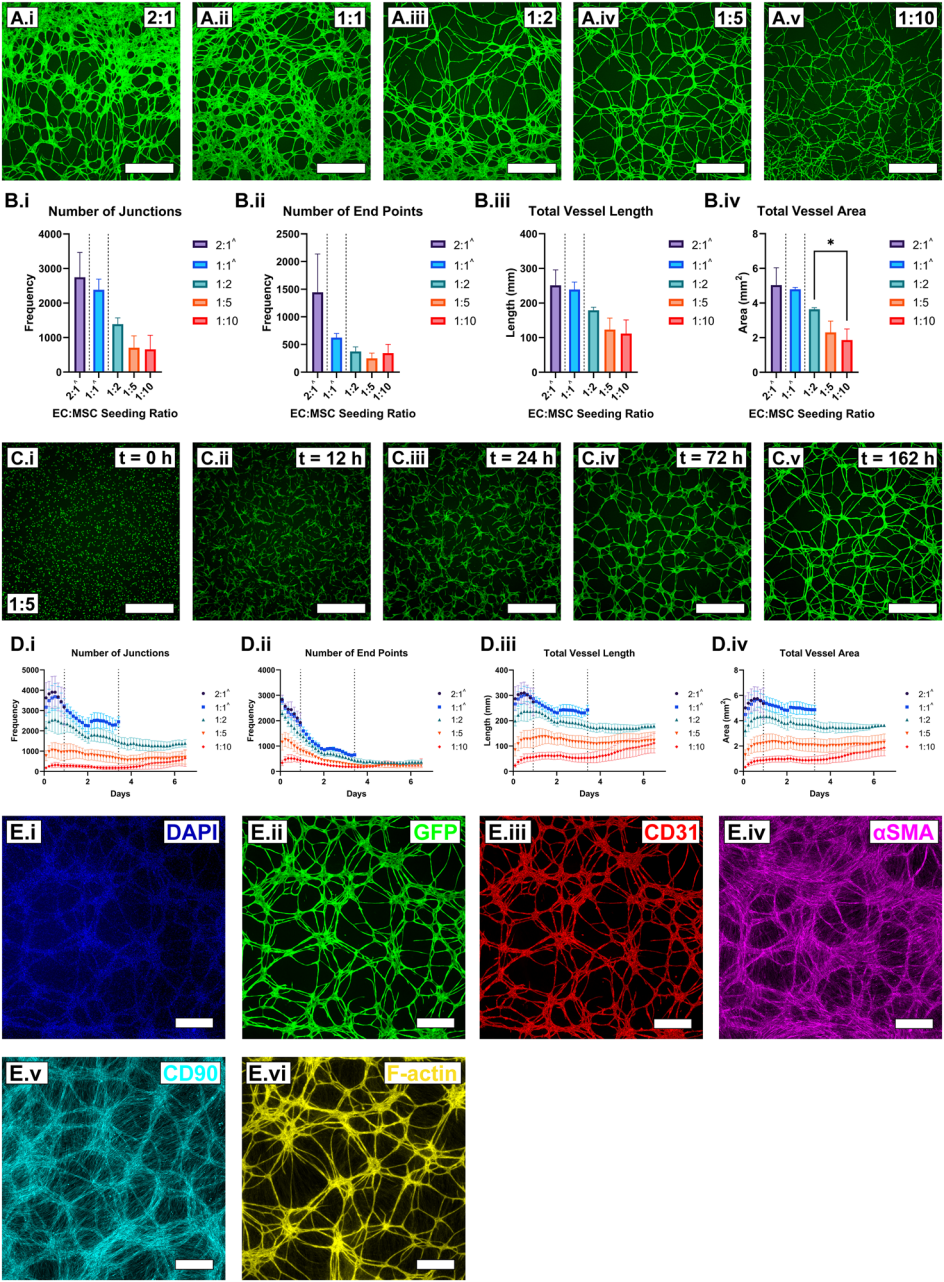
GFP‐hAECs and hAD‐MSCs spontaneously form robust and phenotypical 2D microvascular networks with a morphological dependence on cell seeding ratio. A) Fluorescence images of GFP‐hAEC microvascular networks formed after 7‐days culture in complete vascular cell media, with GFP‐hAEC:hAD‐MSC seeding ratios of 2:1 (*i*), 1:1 (*ii*), 1:2 (*iii*), 1:5 (*iv*) and 1:10 (*v*, scale bars = 1 mm). B) Quantitative analysis of vessel network morphology at day‐7, detailing the number of network junctions (*i*), vessel end points (*ii*), total vessel length (*iii*) and total vessel area (*iv*) within a field of view 3.67 × 3.67 mm (*n* = 3 biological replicas, mean ± standard deviation). Results were compared via an ordinary one‐way ANOVA followed by a Tukey multiple comparisons test (α = 0.05). *p* > 0.05 (ns), *p* ≤ 0.05 (^*^), *p* ≤ 0.01 (^**^), *p* ≤ 0.001 (^***^) and *p* ≤ 0.0001 (^****^). C) Time lapse imaging of GFP‐hAECs in 7‐day vasculogenesis co‐culture with a seeding ratio of 1:5 (scale bars = 1 mm). D) Quantitative analysis of every tenth time‐lapse image (47 of 478 total images) over the entire 7‐day culture period with graphs depicting the number of network junctions (*i*), vessel end points (*ii*), total vessel length (*iii*) and total vessel area (*iv*) within a field of view 3.67 × 3.67 mm (n = 3 biological replicas, mean ± standard deviation). E) Immunocytochemical staining panel of a 1:2 seeding ratio sample after 7‐days culture detailing the nuclear counter stain DAPI (*i*), endogenous GFP expression (*ii*), and detection of CD31 (*iii*), αSMA (*iv*), CD90 (*v*, false colored cyan) and F‐actin (*vi*, false colored yellow, scale bars = 500 µm). Images i–iv, v and vi represent three independent replicate cultures. At times, the 2:1 and 1:1 seeding ratio conditions detached from culture surfaces. The minimum time directly before which a culture commenced detachment (2:1, 22‐h; 1:1, 82‐h; depicted as dotted lines in 1.B and D) was used for the analysis of that condition and therefore was not statistically compared to the cultures which remained attached for the culture duration. EC: endothelial cell, MSC: mesenchymal stem cell, DAPI: 4′,6‐diamidino‐2‐phenylindole, GFP: green fluorescent protein, CD31: cluster of differentiation 31, αSMA: alpha smooth muscle actin, CD90: cluster of differentiation 90, F‐actin: filamentous actin.

The ability of these two cell types to spontaneously self‐assemble into vascular networks was investigated by simultaneously seeding GFP‐hAEC and hAD‐MSCs onto uncoated tissue culture polystyrene at a density of 187500 total cells cm^−2^ with GFP‐hAEC:hAD‐MSC ratios of 2:1, 1:1, 1:2, 1:5 and 1:10 in complete vascular cell media for 7‐days (**Figure** **1**A; Video S1, Supporting Information). Cultures with seeding ratios of 2:1 and 1:1 were observed to commence vessel network formation, however, detached from plates prematurely as early as 22‐ and 82‐h post‐seeding, respectively. Cultures with seeding ratios 1:2, 1:5 and 1:10 formed robust vessel networks which sustained attachment throughout the entire 7‐day culture period (Figure 1A.iii–v). The number of discontinuities in vessel networks after 7‐days culture was observed to increase with decreasing proportion of GFP‐hAECs in the cell seeding mixture (Figure 1A.v).

At culture termination, the number of end points, number of junctions, total vessel length and total vessel area generally decreased with smaller GFP‐hAEC:hAD‐MSC seeding ratios, which displayed a greater average number of end points than the 1:5 seeding ratio (Figure 1B.ii). The time points used for quantification of the 2:1 and 1:1 ratios, which prematurely detached before the 7‐day culture endpoint, were defined as the earliest detachment time of any replicate of that condition (2:1, t = 22 h; 1:1, t = 82 h). These conditions (2:1 and 1:1) should not be directly compared with those which sustained the entire 7‐day culture period (1:2, 1:5 and 1:10).

Time‐lapse microscopy supplemented with image analysis was used to quantitatively examine number of vessel network junctions and endpoints as well as total vessel area and length over a 7‐day culture period (Figure 1C,D; Figure S3, Supporting Information). All four of these metrics demonstrated a rapid increase in magnitude, reaching a maximum at ≈12 h post seeding. After which, these properties began decreasing as individual endothelial cells joined together to form microvascular networks, stabilizing after ≈4‐days in culture (Figure 1C). Post 4‐days culture, the 1:10 seeding ratio culture condition exhibited a further gradual increase in all four measured vessel metrics, which coincided with an observed increase in angiogenic‐like sprouting from established GFP‐expressing vessel‐like structures from day‐4 up to day‐7 (Figure S4, Supporting Information). Angiogenic‐like sprouting was observed from existing vessel‐like structures throughout culture (Figure S5A, Supporting Information) and vessel‐like structures were also observed to merge end‐to‐end (Figure S5B, Supporting Information). No observable signs of abnormal cell function resulting from phototoxicity during timelapse imaging for the length of the 7‐day culture period were observed, when compared to non‐imaged cultures.

At day‐7, 2D vasculogenesis cultures were fixed and immunocytochemically stained for the detection of phenotypical human vascular networks markers (Figure 1E; Figures S6 and S7, Supporting Information). DAPI nuclear staining was observed in nuclei‐like structures through the culture plate and GFP was detected exclusively in vessel‐like structures (Figure 1E.i,ii). Staining for the expression of CD31 was observed exclusively on vessel‐like structures, with staining most intense at cell‐cell junctions (Figure 1E.iii). CD90 staining was observed in cells lining the bottom of the plate and cells lining vessel‐like structures (Figure 1E.iv). Staining for the expression of αSMA was observed strongly in cells lining vessel‐like structures and less so in cells attached the bottom of the culture plate (Figure 1E.v). Chemical staining for filamentous actin was observed in vessel‐like structures and comparatively less in cells occupying extravascular space (Figure 1E.vi). At higher magnification, cells positive for αSMA were observed to line and bridge GFP‐positive vessel‐like structures (Figure S8, Supporting Information).

Vasculogenic co‐cultures require hAD‐MSCs to be cultured in media different to manufacturer specifications. To characterise how different media factors effect GFP‐hAECs and hAD‐MSCs, monocultures were examined for metabolic activity in their respective complete and basal culture medias, as well as hAD‐MSC monocultures in complete vascular cell media (Figure S9, Supporting Information). GFP‐hAEC and hAD‐MSC monocultures in their respective complete medias demonstrated an increase in culture metabolic activity up to day‐5 which was maintained to day‐7. Both GFP‐hAEC and hAD‐MSC monocultures in respective basal medias displayed a reduction in culture metabolic activity across the entire 7‐day culture period. hAD‐MSC monoculture metabolic activity was found to increase when cultured in complete vascular cell media compared to complete MSC media. The vasculogenic potential of GFP‐hAECs was then investigated in the absence of complete vascular cell media supplements and the absence of hAD‐MSCs and compared to a 1:5 GFP‐hAEC:hAD‐MSC control in complete vascular cell media (Figure S10, Supporting Information). In the absence of hAD‐MSCs (cultured in complete vascular cell media), GFP‐hAECs formed a confluent monolayer with little evidence of vessel‐like morphology (Figure S10A, Supporting Information). In the absence of vascular cell media supplements, GFP‐hAEC were observed not to attach to the tissue culture polystyrene culture surface (Figure S10B, Supporting Information). In control conditions, vessel network formation proceeded typically (Figure S10C, Supporting Information).

### 2.2 Microfluidic Culture Conditions Support Spontaneous Formation of 3D Fibrin Hydrogel‐Embedded GFP‐hAECs and hAD‐MSCs into Robust and Patent Microvascular Networks

**Figure 2:**
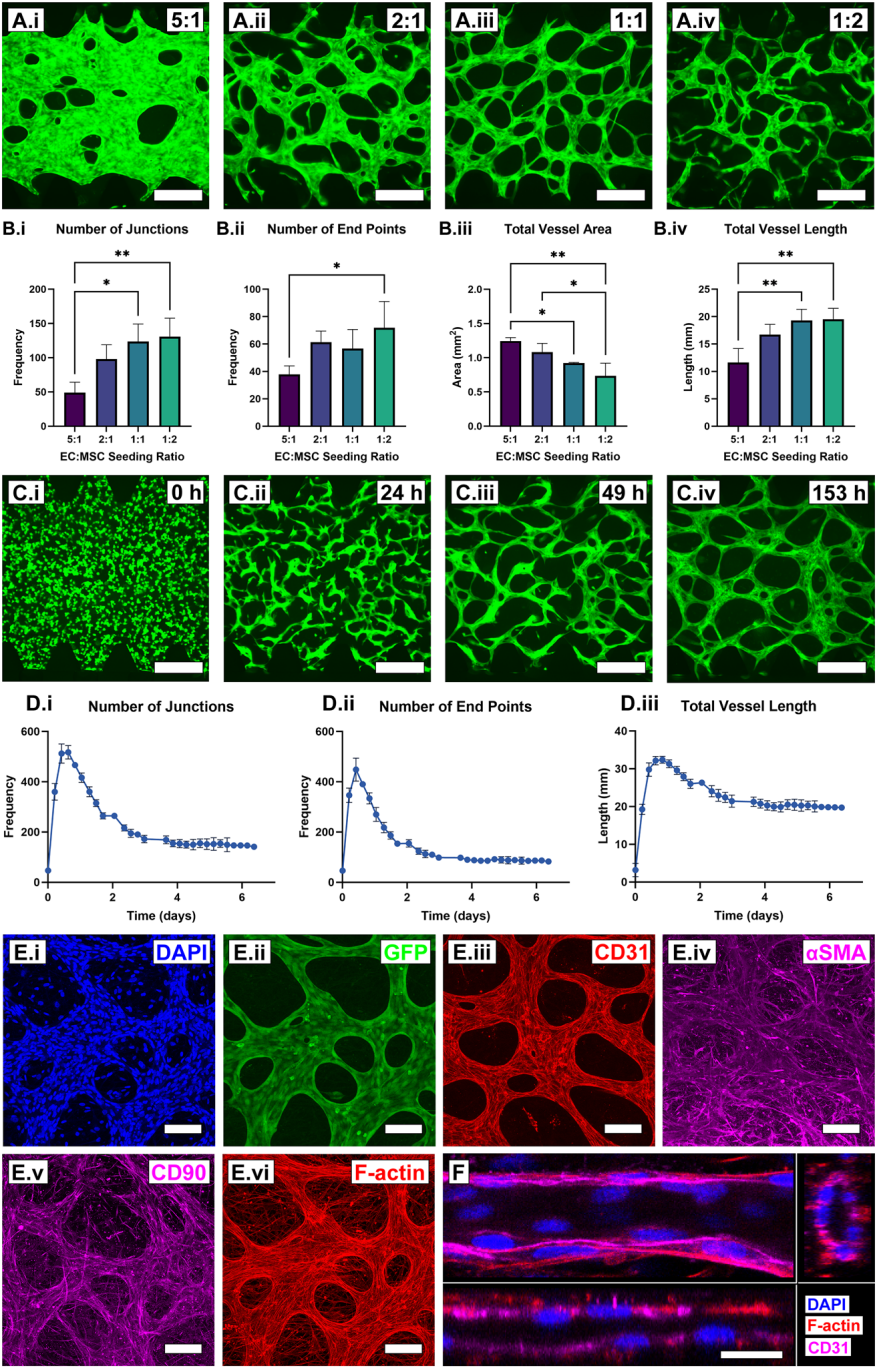
GFP‐hAECs and hAD‐MSCs spontaneously form robust and phenotypical 3D hydrogel‐embedded microvascular networks with a morphological dependence on cell seeding ratio. A. Fluorescence images of GFP‐hAECs as microvascular networks formed after 7‐days fibrin hydrogel‐embedded microfluidic device culture in complete vascular cell media with GFP‐hAEC:hAD‐MSC seeding ratios of 5:1 (*i*), 2:1 (*ii*), 1:1 (*iii*) and 1:2 (*iv*, scale bars = 300 µm). B) Quantitative analysis of vessel network morphology detailing the number of network junctions (*i*), vessel end points (*ii*), total vessel length (*iii*) and total vessel area (*iv*) within a 1.33 × 1.33 mm field of view (n = 3 biological replicas, mean ± standard deviation). Results were compared via an ordinary one‐way ANOVA followed by a Tukey multiple comparisons test (α = 0.05). *p* > 0.05 (ns), *p* ≤ 0.05 (^*^), *p* ≤ 0.01 (^**^), *p* ≤ 0.001 (^***^) and *p* ≤ 0.0001 (^****^). C) Time lapse imaging of GFP‐hAECs in 7‐day vasculogenesis co‐culture with a seeding ratio of 1:1 after 0 (*i*), 24 (*ii*), 49 (*iii*) and 153 h (*iv*) post‐seeding (scale bars = 300 µm). D) Quantitative analysis of every tenth time‐lapse image (28 of 274 images total) of the entire 7‐day culture period, with graphs depicting the number of network junctions (*i*), vessel end points (*ii*) and total vessel length (*iii*) within a 1.33 × 1.33 mm field of view (mean ± standard deviation). E) Immunocytochemical characterization of a 1:1 seeding ratio sample after 7‐days culture, detailing the nuclear counter stain DAPI (*i*), endogenous GFP expression (*ii*), and detection of CD31 (*iii*), αSMA (*iv*), CD90 (*v*) and F‐actin (*vi*, scale bars = 100 µm). F) DAPI nuclear counter stain, CD31 and F‐actin sample cross sections demonstrating vessel patency (scale bar = 20 µm). Images (E.i, ii, vi and F), (E.iii and iv) and v represent three independent replicate cultures. EC: endothelial cell, MSC: mesenchymal stem cell, DAPI: 4′,6‐diamidino‐2‐phenylindole, GFP: green fluorescent protein, CD31: cluster of differentiation 31, αSMA: alpha smooth muscle actin, CD90: cluster of differentiation 90, F‐actin: filamentous actin.

Informed by the optimized ratios deduced from 2D culture experiments (Figure 1A,B), the ability of GFP‐hAECs and hAD‐MSCs to spontaneously form robust vessel networks embedded in 3D fibrin hydrogels was investigated using GFP‐hAEC:hAD‐MSC seeding ratios of 5:1, 2:1, 1:1 and 1:2, (**Figure** **2**A), within previously validated microfluidic device configurations (Figure S11, Supporting Information).^[^ ^25^, ^26^, ^27^ ^]^ Total vessel length, number of junctions and number of endpoints were all found to increase with decreasing GFP‐hAEC:hAD‐MSC seeding ratio (Figure 2B.i‐ii, iv), however, total vessel areas was found to decrease proportionally with decreasing GFP‐hAEC:hAD‐MSC seeding ratio (Figure 2B.iii). Vessel diameter was also qualitatively observed to decrease with decreasing GFP‐hAEC:hAD‐MSC seeding ratio (Figure 2A). Vessel morphology was observed to be consistent across the entire length of the culture compartment (Figure S12, Supporting Information).

When analysed via time‐lapse microscopy, with imaging intervals every 30 min over a 7‐day period (Figure 2C; Videos S2 and S3, Supporting Information), the 1:1 seeding ratio condition demonstrated an increase in number of junctions, number of end points, and total vessel length up to ≈12 h post‐seeding, after which, these values began to decrease and plateau after ≈4‐days and remained constant up to day‐7 (Figure 2D). Total vessel area could not be accurately quantified due to discrepancies and variations in GFP intensities across the surfaces of 3D vessel networks over time.

After 7‐days, 3D vasculogenesis cultures were fixed and immunocytochemically stained for the detection of phenotypical vascular network markers (Figure 2E). Confocal microscopy revealed the detection of CD31 expression in vessel‐like structures, most intensely staining cell‐cell junctions (Figure 2E.iii). CD31 detection was also found in some spherical cells observed inside vessel‐like structures (Figure S13, Supporting Information). αSMA and CD90 detection was observed similarly in cells both lining vessel‐like structures and occupying extra‐vascular space (Figure 2E.iv‐v). F‐actin staining revealed the detection of filament‐like structures, both on and between microvascular networks (Figure 2E.vi). Confocal microscope imaging and 3D reconstruction of vessel‐like structures revealed an outer lumen‐like structure stained positive for the detection of CD31 and F‐actin and a vacant inner lumen absent of staining (Figure 2F). This morphology was found to be consistent throughout the endothelial cell network and the intra‐luminal space was determined to be 9.76 ± 2.11 µm on average in the z‐direction. Cell nuclei were observed to distribute throughout the entire space of a 582 × 582 × 35.6 µm imaging volume encompassing the microvascular network (Figure S14A, Supporting Information). Within this imaged volume, 25.8% of all segmented nuclei (1514 total nuclei) were found to be non‐overlapping (overlap score = 0) with the microvascular network structure (Figure S14B, Supporting Information).

To determine GFP‐hAEC dependency on supplemented media versus hAD‐MSC paracrine‐supported growth, the 3D vasculogenic potential of GFP‐hAECs was assessed in the absence of hAD‐MSCs, the absence of complete vascular cell media supplements and compared to a 1:1 seeding ratio control in complete vascular media (Figure S15, Supporting Information). 3D cultures of GFP‐hAECs (6.5 × 10^6^ cells mL^−1^) in the absence of hAD‐MSCs still formed microvascular networks after 7‐days culture, however, networks displayed discontinuities and GFP‐expressing cell borders were rougher in morphology than typically observed in the presence of hAD‐MSCs (Figure S15A, Supporting Information). When cultured in vascular cell basal media, at a 1:1 seeding ratio, GFP‐hAECs still formed continuous microvascular networks with smooth GFP‐expressing cell borders (Figure S15B, Supporting Information). Microvascular networks formed in vascular cell basal media exhibited an average diameter 19.1 ± 3.72 µm (Figure S14B, Supporting Information), less than control cultures 34.22 ± 1.79 µm (Figure S15C, Supporting Information).

An alternative seeding method was explored to aid the formation of vessel openings between microfluidic device pillars. A suspension of 13 × 10^6^ GFP‐hAECs mL^−1^ was prepared in a hydrogel precursor solution as described in section 2.2.3, pipetted into the device and then immediately aspirated (Figure S16A.i, Supporting Information). A second hydrogel precursor solution was then loaded with 13 × 10^6^ total cells mL^−1^ with a GFP‐hAEC:hAD‐MSC ratio of 1:1 (Figure S16.ii, iii, Supporting Information). This resulted qualitatively in an increased vessel width in between pillars (Figure S16B, Supporting Information) compared to control cultures (Figure S16C, Supporting Information), however, did not support the perfusion of a fluorescently labeled dextran solution throughout vessel networks (Dextran Cascade Blue, 10000 MW, D1976; data not shown).

### 2.3 Microvascular Network Co‐Cultures Enhance hAD‐MSC Adipogenic Differentiation and Subsequent Lipid Production

The adipogenic potential of hAD‐MSCs was initially validated in 2D well plate formats according to manufacturer's protocol for 17‐days total. After 17‐days culture, spherical lipid‐like droplets were visible via brightfield microscopy clustered together and surrounding some cells (Figure S17A.iv, Supporting Information). Lipid morphology was confirmed via staining with the fluorescent lipid‐sensitive dye, LipidTOX (Figure S17A.i–iii, Supporting Information). Adipogenesis cultures also demonstrated the detection of preadipocyte marker PPARG via immunocytochemical staining at day‐17 (Figure S17B,C, Supporting Information).

**Figure 3:**
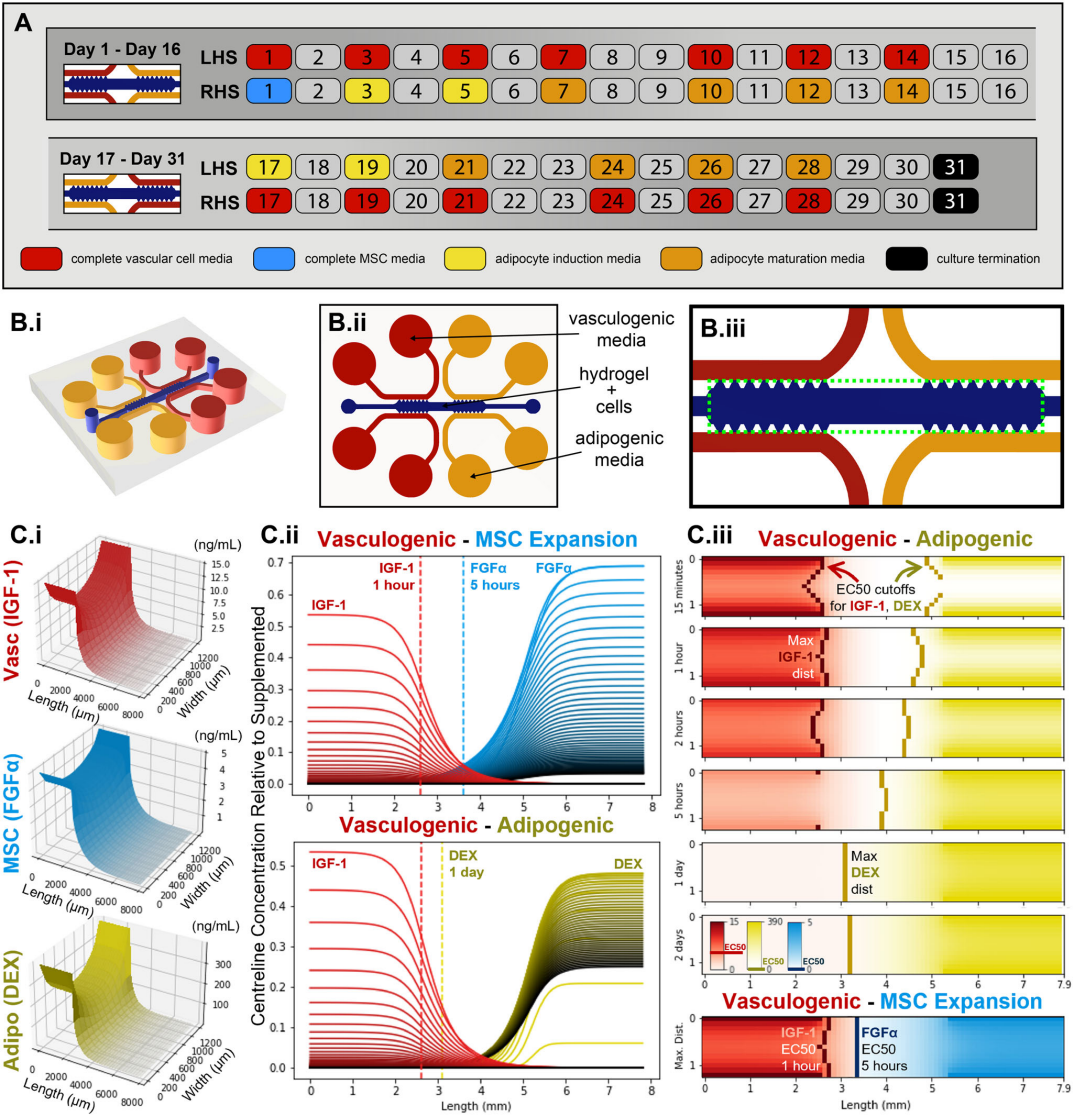
Simulated 3D hydrogel microfluidic co‐culture design for generating counter‐current gradients of vasculogenic and adipogenic factors. A) Gradient device typical feeding strategy depicting each day of a complete 31‐day culture. Both adipocyte induction (light yellow) and maturation media (dark yellow) represent half media exchanges as per section 2.2.5. B) The hydrogel culture compartment (dark blue) is fed either end by adipogenic (yellow) and vasculogenic cell culture media (red; *i*‐*ii*). The culture compartment area (*iii*, green dotted square) is modelled for molecular diffusion. C) The most distribution‐limited vascular (Vasc, red), stromal (MSC, light blue), adipogenic (Adipo, yellow) growth factors were found to be IGF‐1, FGF‐α, and DEX. The maximum simulated concentration (ng mL^−1^) of these factors for each mm^2^ of culture compartment area (1.3 × 7.9 mm) over 2‐days (*i*). Growth factor concentrations across the culture compartment length at mid‐width, relative to supplemented concentrations, where line colour turns darker for each hour of culture up to 2‐days (*ii*). Growth factor concentration heatmaps over culture compartment area and time for the vasculogenic‐adipogenic condition, then maximum distribution of the vasculogenic‐MSC expansion condition at bottom (*iii*). Vertical lines in C.ii and C.iii represent the area where the simulated growth factor concentration is at EC50. Detailed simulations for each growth factor can be found in Figure S18 (Supporting Information), and representative code used to generate Figure 3C can be found in the Supporting Information. LHS: left hand side, RHS: right hand side, IGF‐1: insulin‐like growth factor 1, FGF‐α: fibroblast growth factor acidic, DEX: dexamethasone, EC50: half maximal effective concetration.

Two‐channel microfluidic devices (Figure S11, Supporting Information) were modified to include four channels to support nutrient gradient formation across the device (**Figure** **3**A,B). Numerical modeling of growth factor and small molecule diffusion and decay validated distribution across the length of the device (Figure 3C). The diffusion‐decay of all eight major growth factors in complete MSC media, complete vascular cell media, and adipogenic initiation/maturation culture media were simulated and IGF‐1, FGF‐α, and DEX were found to be the most distribution‐limited factors for each culture media, respectively (Figure S18, Supporting Information).

The fraction of the microfluidic chip's culture area predicted to receive a bioactive concentration of a growth factor (above its EC50) was used to compare growth factor distributions (Figure 3B.ii,iii; Figure S18, Supporting Information). Simulations predicted that vasculogenic factors distributed the shortest distance into the culture compartment; a maximum distribution of 2.6 and 3.1 mm for IGF‐1 or VEGF, respectively. In contrast, stromal growth factors FGF‐α, EGF, or FGF‐β exhibited distributions of 4.2, 5.4, and 7.9 mm (the entire microchip), respectively. Adipogenic factors distribute furthest along the chip, with dexamethasone at 4.7 mm and insulin at 7.9 mm (the entire length of the culture compartment). These predicted distributions were shorter for growth factors with a smaller supplementation‐to‐EC50 concentration ratios or a shorter half‐life (IGF‐1, VEGF, FGF‐α at 24‐, 60‐, and 72‐min). Growth factors with a short half‐life experienced maximum distribution soon after media addition (IGF‐1, VEGF, FGF‐α at 0.5, 1, and 5 h, respectively) and then soon after resided at sub‐bioactive concentrations between media exchanges, as compared to factors with a long half‐life (EGF, FGF‐β, insulin at 1‐, 2‐, and 2‐days, respectively). While a useful design tool, our simulations assume growth factors diffuse and decay similarly as found in liquid cell culture media and do not consider how cellularized fibrin hydrogels release, bind, or otherwise alter growth factor kinetics. Nonetheless, the microfluidic chip culture designs should facilitate the formation of substantial growth factor gradients above and below bioactive concentrations, across the length of the culture compartment, for vasculogenic, stromal growth, and adipogenic factors.

**Figure 4:**
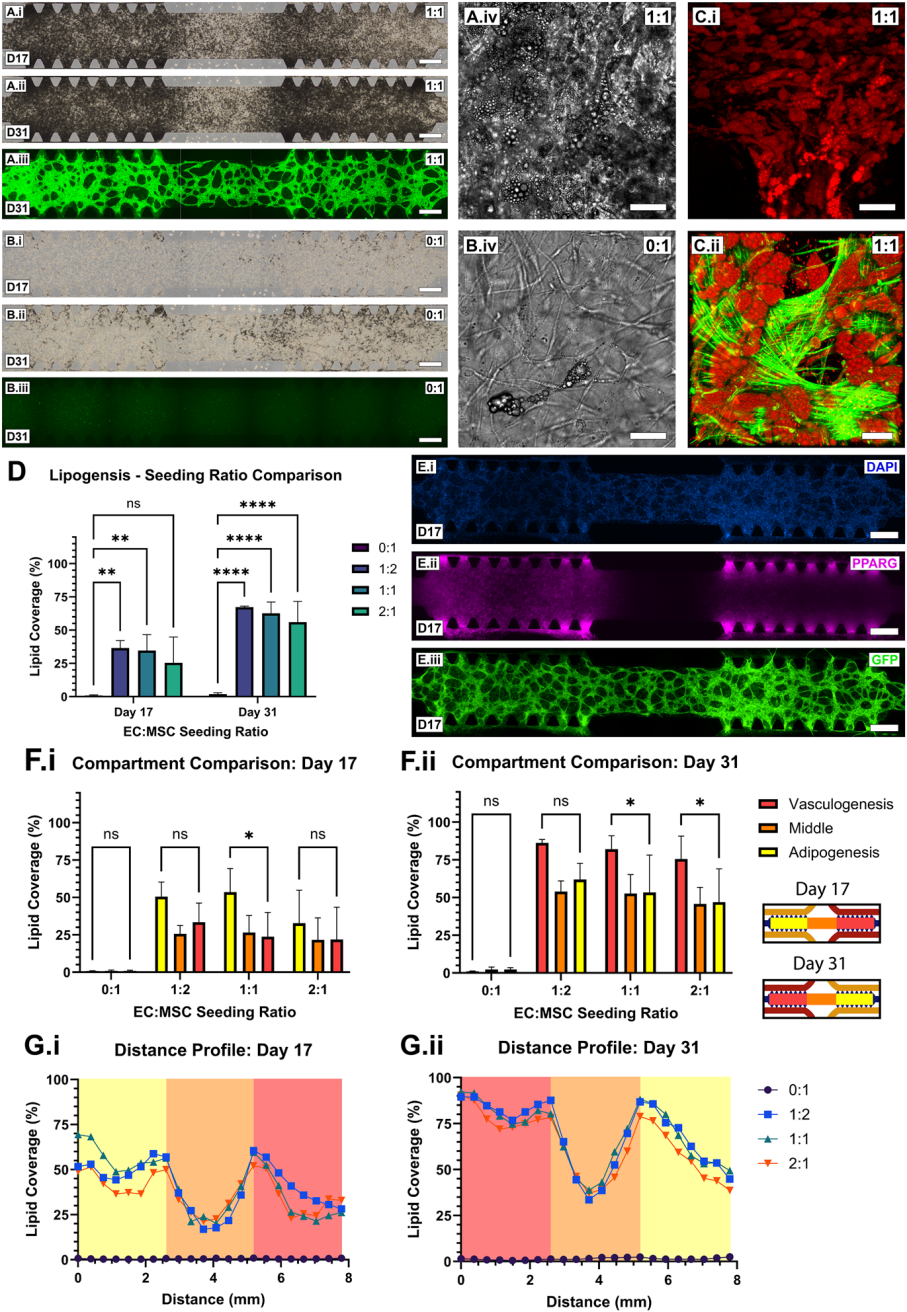
GFP‐hAEC and hAD‐MSC gradient co‐culture supports the co‐formation of microvascular networks and differentiation with vascular network‐enhanced adipogenesis. A) Color bright field microscope images of 1:1 seeding ratio microfluidic device cultures after 17‐ (*i*) and 31‐days (*ii*) gradient culture and green fluorescence after 31‐days culture (*iii*, scale bars = 500 µm). High magnification monochromatic bright field image of the culture compartment (*iv*, scale bar = 50 µm). B) Color bright field microscope images of 0:1 seeding ratio microfluidic device cultures after 17‐ (*i*) and 31‐days (*ii*) gradient culture and green fluorescence after 31‐days culture (iii, scale bars = 500 µm). High magnification monochromatic brightfield image of the culture compartment (*iv*, scale bar = 50 µm). C) LipidTOX staining of 1:1 seeding ratio cultures at day‐31 at low magnification (*i*, scale bar = 50 µm) and high magnification with F‐actin (green) and GFP (*ii*, scale bar = 30 µm). D) Quantitative analysis of lipid coverage across the culture compartment at days‐17 and ‐31 comparing seeding ratios of 0:1, 1:2, 1:1, 2:1 (n = 3 biological replicas, mean ± standard deviation). Results were compared via an ordinary two‐way ANOVA followed by a Tukey multiple comparisons test (α = 0.05). E) Immunocytochemical detection of 1:1 co‐cultures at day‐17 for PPARG with images depicting the nuclear counter stain DAPI (*i*), PPARG (*ii*) and GFP‐hAECs (*iii*, scale bars = 500 µm). F) Percentage lipid coverage localized to each third of the culture compartment defined as vasculogenesis, middle and adipogenesis (inset). Seeding ratios of 0:1, 1:2, 1:1 and 2:1 were investigated at days‐17 and ‐31 of culture (n = 3 biological replicas, mean ± standard deviation). Results were compared via an ordinary one‐way ANOVA followed by a Tukey multiple comparisons test (α = 0.05). *p* > 0.05 (ns), *p* ≤ 0.05 (^*^), *p* ≤ 0.01 (^**^), *p* ≤ 0.001 (^***^) and *p* ≤ 0.0001 (^****^). G) Lipid coverage was similarly quantified in 370 µm increments across the entire length of the culture compartment at day‐7 and ‐31 of culture and plotted for seeding ratios of 0:1, 1:2, 1:1 and 2:1 (*n* = 3 biological replicas, mean). EC: endothelial cell, MSC: mesenchymal stem cell, D: day, F‐actin: filamentous actin, DAPI: 4′,6‐diamidino‐2‐phenylindole, PPARG: peroxisome proliferator‐activated receptor gamma, GFP: green fluorescent protein.

After 17‐days of gradient culture (**Figure** **4**A), a distribution of spherical lipid‐like droplets was qualitatively observed across the device culture compartment using color brightfield microscopy (Figure 4.A.i; Figure S19, Supporting Information). The appearance of lipid‐like droplets was more apparent in GFP‐hAEC and hAD‐MSC co‐cultures of all ratios (1:2, 1:1 and 2:1) when compared to the hAD‐MSC monoculture condition (Figure 4B). After 31‐days culture (following media inversion at day‐17), the abundance and density of lipid‐like droplet in culture continued to increase (Figure 4A.ii,iii). Fluorescent lipid staining and confocal microscope imaging confirmed the presence of lipid droplets throughout the culture chamber (Figure 4C; Figure S20, Supporting Information). Quantitative analysis of color brightfield images revealed a lipid coverage of 67.4 ± 0.07%, 62.67 ± 8.52% and 56.1 ± 15.6% in the 1:2, 1:1 and 2:1 co‐culture conditions, respectively; compared to 1.86 ± 1.09% in the hAD‐MSC mono‐culture condition (Figure 4D). Additionally, immunocytochemical characterization revealed the detection of PPARG after 17‐days of culture (Figure 4E; Figure S21, Supporting Information).

When quantifying lipid coverage per culture compartment (Figure 4F), a significant difference between the adipogenesis and vasculogenesis sides of the device after 17‐days was observable in the 1:1 co‐culture condition (Figure 4F.i). After 31‐days culture, significant differences in lipid coverage were observed in both 1:1 and 2:1 conditions and not in the 1:2 condition (Figure 4F.ii). There was found to be no significant difference in lipid coverage between the adipogenesis and vasculogenesis sides of the device for the 0:1 monoculture condition at either day‐17 or day‐31. This distribution of lipid coverage across the length of the culture chamber was found by quantitative image analysis to be relatively constant across the first third of the device, demonstrated a symmetrical dip in the middle third and decreased in the final third for all co‐culture ratios at both days‐17 and ‐31 post‐seeding (Figure 4G). The monoculture condition was consistently distributed in lipid coverage along the length of the culture compartment at both days‐17 and ‐31 post‐seeding.

### 2.4 Interrogation of Vessel‐Mediated Lipogenesis

**Figure 5:**
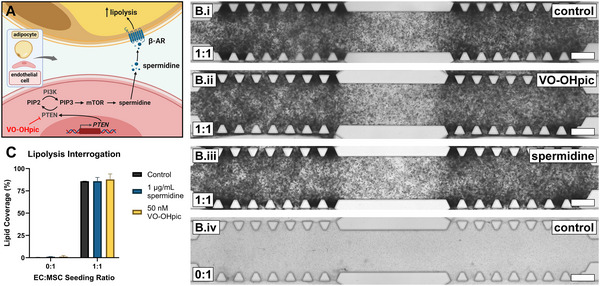
Proven inducers of lipolysis do not inhibit lipid formation in GFP‐hAEC and hAD‐MSC gradient co‐cultures. A) A schematic representing the interactome of vascular PTEN‐mediated lipolysis proposed by Monelli et al,^[^ ^4^ ^]^ the PTEN‐inhibiting effect of VO‐OHpic and lipolysis‐enhancing effect of spermidine. Created with BioRender.com. B) Monochromatic brightfield images of day‐31 1:1 seeding ratio cultures without inhibiting supplements (*i*) with 50 nM VO‐OHpic (*ii*) and 1 µM spermidine (*iii*); and 0:1 seeding ratio cultures without inhibiting supplements (*iv*, scale bars = 500 µm). C) Quantitative analysis of lipid coverage across the culture compartment after 31‐days gradient culture comparing seeding ratios of 0:1 and 1:1 (*n* = 3 biological replicas for spermidine and for VO‐OHpic, *n* = 2 control replicates, mean ± standard deviation). β‐AR: beta‐adrenaline receptor, PI3K: phosphoinositide 3‐kinase. PIP2: phosphatidylinositol 4,5‐bisphosphate, PIP3: phosphatidylinositol (3,4,5)‐trisphosphate, mTOR: mammalian target of rapamycin, PTEN: phosphatase and tensin homolog, VO‐OHpic: hydroxyl(oxo)vanadium 3‐hydroxypiridine‐2‐carboxylic acid, EC: endothelial cell, MSC: mesenchymal stem cell.

Modulation of lipogenesis in gradient co‐cultures was investigated using know inhibitors of adipogenesis and inducers of lipolysis, VO‐OHpic and spermidine.^[^ ^4^, ^28^ ^]^ To ensure VO‐OHpic and spermidine had no influence on vasculogenic cells, monocultures of GFP‐hAECs and hAD‐MSCs were tested for metabolic activity in the presence of respective growth media supplemented with 0, 5, 50 and 500 nM VO‐OHpic and 0, 0.1, 1 and 10 µM spermidine. Culture metabolic activity of either cell type was not significantly influenced by any of the investigated molecule concentrations (Figure S22, Supporting Information). Both 1:1 co‐cultures and 0:1 monocultures were conducted individually in the presence of 1 µM spermidine, 50 nM VO‐OHpic and in their absence (**Figure** **5**A; Figure S23, Supporting Information). The 1:1 and 0:1 control conditions was conducted only with two biological replicas due to a technical error. All remaining conditions encompassed three biological replicates. Captured monochromatic brightfield images of 1:1 co‐cultures at day‐31 demonstrated a typical dark shade of spherical droplets distributed throughout the culture compartment of control and similarly in 1 µM spermidine and 50 nM VO‐OHpic conditions (Figure 5B.i–iii). Brightfield images of all monocultures conditions demonstrated a consistent light shade with some small and sparse dark regions (Figure 5B.iv). Co‐cultures were determined via quantitative image analysis to demonstrate a lipid coverage of 85.9 ± 0.07%, 85.9 ± 4.23% and 87.9 ± 6.15% for control, 1 µM spermidine and 50 nM VO‐OHpic conditions, respectively (Figure 5C). No difference was observed between the control culture condition and either of the supplemented culture conditions. Similarly, monocultures demonstrated a lipid coverage of 0.25 ± 0.28%, 1.03 ± 0.25%, and 1.25 ± 1.34% for control, 1 µM spermidine and 50 nM VO‐OHpic conditions, respectively and no difference was observed between the control culture condition and either of the supplemented culture conditions.

## 3 Discussion

For the first time in scientific literature, we detail a thorough optimization and validation of the culture conditions required to construct highly reproducible microvascular networks from green fluorescent protein‐expressing hTERT immortalised human aortic endothelial cells (GFP‐hAECs) co‐cultured with hTERT immortalized adipose‐derived mesenchymal stem cells (hAD‐MSCs). While numerous studies have demonstrated the utility of primary MSCs and ECs as models of vasculogenesis, immortalized cell lines exhibit distinct advantages for overcoming batch‐to‐batch variability and limited in vitro lifespan, both of which are detrimental to the reproducibility of microvascular network studies and human preclinical cell culture models.

The vasculogenic ability of GFP‐hAEC and hAD‐MSCs was initially investigated and optimized with common and established 2D monolayer culture technique with the motivation for informing the design of 3D hydrogel‐embedded microfluidic cultures protocols. GFP‐hAEC and hAD‐MSC seeding ratio was demonstrated to be a critical determinant of vascular network morphology. Seeding ratios of 2:1 and 1:1 resulted in entire culture monolayer detachment from the culture surface before day‐7 (Video S1, Supporting Information). It is hypothesized that were the 2:1 and 1:1 seeding ratio conditions able to continue to culture day‐7, continued cell expansion and vessel merging (reduction in junctions) may have been observed. It is suspected that the formation of microvascular networks introduce tension on the cell monolayer, which is proportional to the number of endothelial cells. Additionally, these higher endothelial cell seeding ratios resulted in larger vessel junctions and reduced extravascular space (Figure 1A.i‐ii), a morphology less consistent with in vivo human capillary networks.^[^ ^29^ ^]^ The latter phenomenon was also evident, however, less so, in the 1:2 condition (Figure 1A.iii). The lowest GFP‐hAEC and hAD‐MSC seeding ratio studied (1:10) resulted in the lowest vessel area and total vessel length (Figure 1B.iii‐iv), almost certainly due to the reduced number of seeded endothelial cells. Interestingly, after day‐4, the 1:10 GFP‐hAEC:hAD‐MSC condition demonstrated significant angiogenesis toward vacant extravascular space, evident both qualitatively in images and quantitatively by an uptick in all analyzed vessel metrics (Figure 1D; Figure S4, Supporting Information). This knowledge is pertinent for future studies examining angiogenesis post‐establishment of stable microvascular networks. The 1:5 condition demonstrated a maximum network density with non‐enlarged junctions while maintaining surface adhesion throughout the 7‐day culture period (Figure 1A.iii,B; Video S1, Supporting Information) and was therefore determined to be the optimal cell seeding ratio for 2D vasculogenesis. While both cell types were presumed to be homogeneously distributed during the seeding process, unevenness in surface attachment distribution was observed to result in a distribution in network density across the culture surface. This variability was accounted for by sampling fields of view across three technical replicates per each of three biological replicates.

Microvascular network phenotype was assessed against a panel of markers consisting of the endothelial cell marker, CD31; the pericyte‐like cell marker, αSMA; the MSC marker, CD90; and the filamentous cytoskeletal protein marker, F‐actin. As expected, the detection of CD31 was abundant in regions consistent with hAEC‐GFP expression and vessel‐like morphology (Figure 1E.ii,iii). At high magnification, localization of CD31 detection appeared most abundant at cell‐cell junctions in vessels, consistent with in vivo human vessel phenotype.^[^ ^30^ ^]^ Detection for αSMA was observed across the culture surface, however, appeared most abundant in cells surrounding vessels (Figure 1E.iv). At high magnification, αSMA positive cells can be seen lining the outside of vessels, consistent with in vivo non‐quiescent pericyte‐like behavior (Figure S8B.i, Supporting Information)^[^ ^31^ ^]^ and additionally appear to be forming bridges across vessels (Figure S8B.iii, Supporting Information), potentially as a precursor to angiogenesis and end‐to‐end anastomosis. Detection for CD90 was observed in cells throughout the culture with no distinct localizations to specific regions. CD90‐positive cells on the bottom of the culture surface appear to adopt an aligned morphology (Figure 1E.v). The retention of a CD90 positive phenotype indicates a retention toward multipotency, among other traits.^[^ ^32^ ^]^

Time‐lapse microscopy and image analysis was utilized to quantify vasculogenesis and characterize this morphogenetic process. Imaging typically commenced 2 h after cell seeding, to ensure the movement of the microscope stage did not influence cell attachment, Additionally, a single technical replicate commencing imaging directly after seeding was conducted in isolation to confirm morphogenetic behavior during the 0–2 h period (Figure S3, Supporting Information). All seeding ratios demonstrated a similar trend in total vessel area, total vessel length, number of junctions and number of end points, which was a sharp increase in magnitude from seeding to a peak at ≈12 h post‐seeding (Figure 1D). This was then followed by a decrease in magnitude until approximately day‐3 and followed by stabilization until the termination of culture at day‐7. It is therefore hypothesized from these observations that in vitro vascular morphogenesis occurs in three distinct morphogenetic phases: 1) the formation of discrete and discontinuous early vessel‐like structures (0–0.5 days), 2) anastomosis of discrete early vessels toward a continuous network (0.5–4 days) and 3) maturation and readjustment of a continuous vessel network (4–7 days). In low‐endothelial cell number cultures (e.g., 1:10 condition), angiogenesis can also be encompassed in this third developmental phase (Figure S4, Supporting Information). These findings also agree with the mechanisms reviewed by Venkatakrishnan et al, which describe the *de novo* assembly of discrete endothelial cells into the first blood vessels of the early embryo.^[^ ^33^ ^]^ Both angiogenic and end‐to‐end anastomotic‐like processes were observed via time lapse microscopy to occur in these cultures (Figure S5, Supporting Information), highlighting the utility of this system, not only for predictably studying vasculogenesis in vitro, but also angiogenesis and anastomosis. The removal of spent media and the addition of fresh media was observed to coincide with a small uptick in all measured vessel network properties (Figure 1D; Figure S3, Supporting Information), most likely due to the refreshing of active vasculogenic factors in the culture volume. The ability to accurately and quantitively assess and predict microvascular tissue development is of tremendous importance toward industrial standardization of preclinical vascular models and therapeutic screening platforms. Furthermore, the ability to quantify in vitro tissue development in real time would allow informed and timely intervention to rescue, terminate, feed, differentiate or harvest a culture, improving process efficiency and reducing running costs for regenerative therapy manufacturing.

GFP‐hAEC in vitro vasculogenesis was shown to critically rely on both the presence of hAD‐MSCs in culture and the inclusion of an endothelial cell growth factor cocktail in media (Figure S10, Supporting Information). It was hypothesized that GFP‐hAEC vasculogenesis could potentially proceed in the absence of an endothelial cell growth factor cocktail (and rely purely on paracrine signaling from hAD‐MSCs) or in the absence of supporting hAD‐MSCs, to aid simplicity and reduce culture cost. In the absence of the endothelial cell growth factor cocktail, GFP‐hAECs did not attach to the culture substrate post‐seeding (Figure S10B, Supporting Information). Additionally, in the absence of supporting hAD‐MSCs (in the presence of endothelial cell growth factor cocktail), GFP‐hAECs simply attached and continued expanding as an overconfluent monolayer (Figure S10A, Supporting Information). Interestingly, the metabolic activity of hAD‐MSC cultures in complete endothelial cell media at days‐5 and ‐7 of cultures was found to be more than double that in complete MSC media (Figure S9, Supporting Information), potentially due to the presence of IGF‐1 in complete vascular cell media, which is a known promoter of MSC proliferation.^[^ ^34^ ^]^ IGF‐1 potentially contributes to the activity of hAD‐MSCs in vasculogenic co‐culture, which was typically conducted in complete vascular cell media.

2D vasculogenesis co‐culture technology was translated to a fibrin hydrogel‐supported 3D culture system within a commonly adopted microfluidic device configuration (Figure S11, Supporting Information).^[^ ^25^, ^26^, ^27^, ^35^ ^]^ The small molecule, aprotinin, was included in the hydrogel formulation to prevent the degradation of fibrin due to its antifibrinolytic properties.^[^ ^36^ ^]^ Like 2D findings, GFP‐hAEC and hAD‐MSC co‐cultures exhibited stable vessel network formation after 7‐days culture in complete endothelial cell media and similarly exhibited a cell seeding ratio dependence on vascular network morphology (Figure 2A,B). At high seeding ratios (5:1, 2:1) vessel junctions increased in size and vessels merged into an agglomerated structure, subsequently causing an increase in vessel area and decreases in vessel length, number of junctions and number of end points (Figure 2A.i,ii). As the 2:1 (and 1:1) 2D seeding ratio conditions prematurely detached from culture surfaces before day‐7, a direct comparison with the same ratios in 3D cultures cannot be made. At the lowest seeding ratio (1:2), vessel networks were highest in length, junctions and end points, however, exhibited discontinuities throughout the network (Figure 2A.iv). A seeding ratio of 1:1 resulted in network continuity with maximum length, junctions and end points (Figure 2A.iii) and was determined to be the ideal seeding ratio for creating stable and robust vessel networks from these immortalized cell types suspended in fibrin hydrogels. Of note is the small magnitude of standard deviations of vascular metrics for both 3D and 2D vasculogenesis systems (Figures 1B and 2B), indicating high reproducibility.

Consistent with 2D morphogenetic study outcomes, vessel networks formed in 3D culture environments (1:1 seeding ratio) demonstrated a rapid increase in junctions, vessel length and end points (0–0.5 days), followed by a decrease (0.5–3.5 days) and plateau (3.5–7 days). Unlike the 1:10 seeding ratio in 2D, no angiogenesis was observed during the plateau phase (Figure 2D). An identical immunocytochemical marker panel demonstrated similar detection of CD31 localized to vessel cell junctions, αSMA and CD90 in cells both on and between vessel and F‐actin on the cytoskeleton of cells in vessels and in the extravascular space (Figure 2E). Furthermore, confocal microscope cross‐sectional images confirmed vessel patency (Figure 2F). Unlike 2D cultures, αSMA was detected with similar intensity in cells occupying the extracellular space and cells lining vessels (Figures 1E.iv and 2E.iv). This difference is potentially a result of the increased dimensionality of the culture or the mechanical properties of the fibrin hydrogel.^[^ ^37^ ^]^

Like 2D cultures, vasculogenesis was investigated in the absence of hAD‐MSCs and in 3D co‐cultures with EC basal media (without a growth factor cocktail). Interestingly, unlike 2D results, vascular networks formed in both conditions (Figure S15, Supporting Information). In the absence of hAD‐MSCs, endothelial cells still self‐assembled into vessel networks, however, network quality was poor, exhibited by discontinuities throughout the network and rough vessel borders (Figure S15A, Supporting Information), likely a result of both absent paracrine signaling and the physical stabilizing effects of pericyte‐like cells, in the form of hAD‐MSCs. In the absence of endothelial cell media growth factor cocktail, GFP‐hAEC and hAD‐MSC co‐cultures still formed continuous vessel networks with tight vessel borders, however, with smaller vessel diameter and junctions compared to those with the growth factor cocktail (Figure S15B, Supporting Information). The influence of fibrin hydrogel biomechanical and biochemical properties should be highlighted here when considering the favorable stabilizing effects of this culture environment. These differences suggest that GFP‐hAECs and hAD‐MSCs provide mutually supportive paracrine factors enabling microvascular network establishment in 3D (but less so 2D). Additionally, these findings suggest vessel and junction diameters (and therefore projected area) could be tuned based on the concentration of growth factors in basal media.

The presence of vessel openings spanning inter‐pillar spacing is a phenomenon sometimes seen in vasculogenic co‐cultures using similar microfluid device configurations with alternative cell types, such as human umbilical vein endothelial cells and lung fibroblasts.^[^ ^26^, ^35^ ^]^ Vessel networks in this study did not form visible or functional openings between pillars (Figure S16C, Supporting Information), whose closure was confirmed via addition of fluorescently labeled dextran (data not shown). To encourage vessel openings between pillars, an approach similar to that of Wan et al^[^ ^25^ ^]^ was adopted, whereby, 13 × 10^6^ GFP‐hAECs mL^−1^ (no hAD‐MSCs) were seeded into the device and immediately aspirated, leaving a suspension of entirely endothelial cells between the pillars (Figure S16A, Supporting Information), after which, the 1:1 co‐culture was used to fill the culture compartment completely. While this approach aided in providing broader vessel structures between pillars (Figure S16B, Supporting Information), these structures remained closed to the neighboring media channel and did not allow the passage of fluorescently labeled dextran (data not shown). The reason as to why this model did not support typically vessel opening between pillar spacing is unknown. One hypothesis is that mural cell‐like contractility prevents vessel opening, as the down regulation of CD90 in hAD‐MSCs was demonstrated in late passage hTERT immortalized human fibroblasts by Wan et al to inhibit the ability of forming inter‐pillar openings,^[^ ^38^ ^]^ however, this was not actively examined in this study.

The typical 2‐channel microfluidic chip geometry (Figure S11, Supporting Information) utilized in experimental outcomes presented in Figure 2 was modified to included 4‐channels (Figure 3B) for facilitating the distribution of growth factors across the length of the culture compartment and was modeled numerically (Figure 3C; Figure S18, Supporting Information). While numerical modeling provides an approximation of growth factor transport, further experimental validation such as real‐time fluorescent molecule tracking, biosensing or ELISA‐based measurements are required to confirm these predictions. When differentiated using this gradient culture device (Figure 3; Figure S18, Supporting Information), vessel networks and adipocytes were observed to form within the same 3D space (Figure 4A). The adipogen‐rich side of the device was found not inhibit the formation of continuous vascular networks, which were observed throughout the culture compartment after day‐17. Post‐inversion of culture gradients, at day‐31, vessel network morphology was maintained and lipid droplet intensity increased (Figure 4A; Figure S19, Supporting Information), however, full homogeneity of lipid production across the culture compartment was not entirely achieved (Figure 4A.ii,F.ii,G.ii). It is hypothesized that hAD‐MSCs lose their differentiation potential when maintained in a vasculogenic environment for 17‐days.^[^ ^39^ ^]^ One proposed solution could be to reduce the time before inverting the culture environments at day‐17 to prevent such loss of potency. The intensity of lipid production in the presence of microvascular networks was remarkably high (67.4 ± 0.07%) when compared to hAD‐MSC 3D monocultures (1.86 ± 1.09%), consistent with the landmark murine model findings of Rupnick et al.^[^ ^40^ ^]^ The intensity of lipogenesis was surprising given the limited adipogenic potential of hTERT immortalized hAD‐MSCs reported by Masnikov et al.^[^ ^41^ ^]^ While this study validated an adipocyte‐like phenotype using LipidTOX lipid and PPARG immunocytochemical staining, it could benefit from further confirmation of this phenotype and assessment of adipocyte maturity using extensive qPCR and immunocytochemical marker panels (e.g., Pref‐1, FABP4, adiponectin), ELISAs (e.g., leptin), colorimetric lipid quantification assays and assessing adipocyte mitochondrial activity.

While the mechanisms which underpin this vasculo‐adipo relationship have remained poorly understood, Monelli et al reported a mechanism, proposing vessel endothelium aids in suppressing adipose tissue lipolysis.^[^ ^4^, ^42^, ^43^ ^]^ By deleting endothelial *Pten* (phosphatase and tensin homolog) in mice, Monelli and colleagues observed a reduction in bodyweight and adiposity compared to control littermates. They confirmed *Pten* deletion supported sustained phosphoinositide 3‐kinase (PI3K) activity and enhanced polyamine synthesis (specifically spermidine) in endothelial cells, which promoted beta‐adrenaline receptor activity and up regulated lipolysis in *ex vivo* mouse WAT explants, primary mouse adipocytes and in vivo murine models. This led us to investigate whether this PTEN regulated mechanism could regulate lipid production in our human 3D co‐culture model.

VO‐OHpic is a known inhibitor of PTEN, with reported EC50 value of 50 nM^[^ ^44^, ^45^ ^]^ and as described above, spermidine, a polyamine, has been demonstrated to induce lipolysis in primary mouse adipocytes at a concentration of 1 µM.^[^ ^4^, ^46^, ^47^ ^]^ These two molecules at these concentrations were therefore chosen to investigate their ability of inducing lipolysis in our human co‐culture model. The stages of adipogenesis at which these compounds act remains unknown; hence these compounds were supplemented for the entire 31‐day culture period. Surprisingly, supplementation of this co‐culture differentiation model with said compounds resulted in no change in percentage lipid coverage after 31‐days co‐culture (Figure 5; Figure S23, Supporting Information). It is hypothesized that the limited complexity or maturity of this co‐culture model does not completely recapitulate the multifaceted interactions of multicellular murine WAT tissue explants or the maturity of primary adipocytes. Our model also deviates from peripheral adipose tissue anatomy which contains fewer vessels of smaller diameter, more like Figure S15B (Supporting Information).^[^ ^48^, ^49^ ^]^ Additionally, the selected concentrations of the chosen molecules could potentially be below threshold values required to illicit the expected biological responses. The cross‐species differences (murine vs human) when comparing the outcomes of the interactome proposed by the murine model of Monelli et al with the new human in vitro model presented in this research, should also be considered. The experiments presented in Figure 5 are limited in scope to image quantification. Future experimental validation of the PTEN mechanism with this model such as, monitoring changes in signaling pathways via Western blot, qPCR and immunocytochemical staining should be considered.

Inducing the formation of physiologically relevant microvascular networks within developing in vitro tissue constructs is challenging due to the inherent nature of nutrient delivery in modern 2D batch culture approaches, which fail to mimic the complex spatial and temporal delivery seen in vivo. Technology which supports reproducible spatial and temporal control of biochemical growth environments is necessary for the development and subsequent scale up of in vitro tissue and organ products. Microfluidic culture technologies are advantageous as disease modeling and therapeutic screening platforms compared to 3D bioprinted constructs as they are inexpensive to fabricate, inexpensive to operate with small media volumes (<500 µL), mitigate nutrient and oxygen diffusion limitations due to small culture volumes (10 µL), maximize throughput due to low required cell numbers (13,000 total cells per culture) and are amenable to high‐quality in situ imaging techniques.^[^ ^50^ ^]^ Disadvantages include initial random and uncontrolled distribution of cells, which could prevent a true recapitulation of healthy and diseased adipose tissue anatomy; and confined culture geometry, which could limit growth and maturation of the system beyond 31‐days culture and hinder scale up.^[^ ^51^ ^]^ By designing 2D, 3D hydrogel, and microfluidic co‐culture technology to spatiotemporally control vasculogenesis, we deliver a highly reproducible culture system using immortalized human cell lines. We then apply our 3D hydrogel microfluidic model to generate counter‐current media gradients, demonstrating a new approach for the co‐development and integration of MSC‐derived adipocytes and patent microvascular networks, within the same 3D tissue space. This serves as a platform technology for the scale up of vascularized mature connective tissue co‐cultures, potentially adopting 2D and 3D channel arrays to control growth and differentiation in 3D, providing future avenues in vascular tissue models and for disease and biotherapeutic screening.

## 4 Conclusion

Biomanufacturing of vascularized connective tissue has broad applications toward tissue implant engineering, drug discovery, and cultivated meat. However, vasculogenic co‐cultures suffer from reproducibility, scale‐up to 3D, and an inability to vascularize and differentiate connective tissue simultaneously. In this study, optimal, robust and phenotypic vascular networks were formed in 2D culture by co‐seeding hTERT immortalized green fluorescent protein‐expressing human aortic endothelial cells (GFP‐hAECs) with hTERT immortalized human adipose‐derived mesenchymal stem cells (hAD‐MSCs) at a density of 187500 total cells cm^−1^ and a seeding ratio of 1:5. GFP‐hAECs and hAD‐MSCs similarly formed robust and phenotypical vascular networks when seeded suspended in 3D fibrin hydrogels at a density of 13 × 10^6^ total cells mL^−1^ with a seeding ratio of 1:1. Time‐lapse microscopy and quantitative image analysis revealed a consistent trend in vessel network junctions, end points and length throughout development, demonstrated by an initial sharp rise, peak, decrease and plateau in morphological properties. Gradient microfluidic culture supported the co‐formation of integrated microvascular networks and functional adipocytes within a single 3D hydrogel structure. Adipogenesis was drastically enhanced in the presence of vessel networks (67.4% lipid coverage) compared to in their absence (1.86% lipid coverage). Supplementation of gradient co‐cultures with 50 nM VO‐OHpic and 1 µM spermidine did not suppress lipogenesis, as previously reported with murine models. This research demonstrates a new in vitro culture system capable of generating reproducible vascularized adipose tissue from existing commercial immortalized human EC‐ and MSC‐lines. This model has significant potential for studying vascular‐adipose crosstalk in human preclinical studies of diabetes, obesity, and for biotherapeutic screening.

## 5 Experimental Section

### Microfluidic Device Design, Fabrication and Preparation

Microfluidic devices were designed using AutoCAD (Autodesk, Inc.) 3D design software (Figure S11, Supporting Information). Silicon SU‐8 master wafers (7‐inch diameter) featuring said designs were then prepared by the Australian National Fabrication Facility Queensland Node. Briefly, a UV mask aligner was used to transfer designs onto a photoresist‐coated silicon master wafer to a feature height of 200 µm.

Silicon master wafers were treated overnight with the vapor of hexamethyldisilizane and then secured in Petrie dishes using Scotch tape. Using SYLGARD 184 Silicone Elastomer Kit (Dow), 10‐parts monomer was added with 1‐part co‐monomer and simultaneously mixed and degassed using a Kurabo KK‐V300SS planetary centrifugal mixer and poured onto the master wafer to a height of ≈5 mm above the wafer. The mixture was then cured at 80 °C for a minimum of 1 h. The cured elastomer was then carefully removed using a scalpel and media reservoirs and cell injection ports were created using 1.25‐ and 4‐mm diameter biopsy punches, respectively. Both elastomer and microscope slides were cleaned with Scotch tape, placed face‐up on the stage of a Tergeo Tabletop Plasma Cleaner and treated with oxygen plasma at 35 W for 40 s. Elastomer and slide were bonded with direct contact before full adhesion was achieved after ≈5 min.

Microfluidic devices were wet autoclave sterilized at 121 °C before being washed of any residue via complete immersion in 70% (w/v) ethanol for a minimum of 1 h. Ethanol was then completely removed from the devices via vacuum aspiration before the devices were washed in triplicate with sterile distilled water (15 230 162). Devices were finally dried overnight in a humidified incubator at 37 °C in 5% (v/v) CO_2_ in air to remove any remaining water from channels.

### Cell Culture and Imaging

This project was supported by The University of Queensland's Human Research Ethics Committee through approval 2021/HE002698: “Tissue culture models for in vitro optimization of engineered biomaterial scaffolds and bioprocess techniques”, as well as The University of Queensland's Institutional Biosafety Committee through approval IBC/602E/ChemEng/2023: “Risk group 2 immortalized cell line activities.”

### Cell Culture and Imaging—*Routine Cell Line Maintenance*

Human telomerase reverse transcriptase (hTERT) immortalized adipose‐derived mesenchymal stem cells (hAD‐MSCs; ASC52telo, SCRC‐4000, American Type Culture Collection ATCC) were routinely maintained as per manufacturer's instructions in MSC Basal Medium (#500‐040) supplemented with Mesenchymal Stem Cell Growth Kit for Adipose and Umbilical‐derived MSCs – Low Serum (#500‐030), herein referred to as “complete MSC media”, and subcultured at a confluence of ≈80%.

hTERT immortalized green fluorescent protein‐expressing human aortic endothelial cells (GFP‐hAECs; TeloHAEC‐GFP, CRL‐4054, ATCC) were routinely maintained as per manufacturers instruction in Vascular Cell Basal Medium (#100‐0303) supplemented with Endothelial Cell Growth Kit – VEGF (#100‐041) and 33 µM phenol red (#999‐001), herein referred to as “complete vascular cell media”, and subcultured at a confluence of ≈80%. Maintenance cultures of both cell types were routinely tested for mycoplasma contamination using MycoStrip – Mycoplasma Detection Kit (rep‐mys‐50).

### Cell Culture and Imaging—*2D Vasculogenesis Culture*

To induce spontaneous formation of vascular networks from endothelial cells (vasculogenesis), hAD‐MSCs were co‐cultured with GFP‐hAECs in a humidified incubator at 37 °C in 5% (v/v) CO_2_ in air. In brief, hAD‐MSCs and GFP‐hAEC maintenance cultures were washed with room temperature Dulbecco's phosphate buffered saline solution (DPBS, 14 190 144, 3 mL/25 cm^2^) before being exposed to 0.05% trypsin‐EDTA solution (25 300 054, 1 mL/25 cm^2^) for approximately 5 min, at room temperature for hAD‐MSCs, and 37 °C for hAECs. Cultures were then neutralized with an equal volume of respective maintenance media, before being centrifuged at 200 × g for 5 min at room temperature. Cell pellets were resuspended and counted via trypan blue exclusion method with a Countess 3 Automated Cell Counter (ThermoFisher Scientific). hAD‐MSCs and GFP‐hAECs were then mixed at cell ratios from 1:10 to 10:1 and centrifuged again at 200 × g for 5 min at room temperature. The cell pellet was resuspended in complete vascular cell media and cells were then seeded at a density of 187500 total cells cm^−2^, based on the optimized works of Evensen et al,^[^ ^52^ ^]^ on Corning 96‐well Clear Flat Bottom Polystyrene TC‐treated Microplates (3598).

### Cell Culture and Imaging—*3D Vasculogenesis Device Culture*

*Reagent preparation*: Fibrinogen type 1‐S from bovine plasma (F8630) stock solution was prepared as per manufacturer instructions at 10 mg mL^−1^ in DPBS before being sterilized using a 0.22 µm syringe filter. Thrombin from bovine plasma (T7513) stock solution was prepared at 100 U mL^−1^ in 0.1% (w/v) bovine serum albumin solution and similarly sterilized. Aprotinin (A1153) was dissolved in DPBS to give a stock solution of 15 U mL^−1^ and sterile filtered.

*Device seeding*: Fibrinogen stock solution was diluted to 6 mg mL^−1^ in DPBS. Suspensions of GFP‐hAECs and hAD‐MSCs were prepared in respective cell maintenance medias and mixed at ratios from 5:1 to 1:2. Cell mixtures were centrifuged at 200 × g for 5 min and resuspended at 26 × 10^6^ cells mL^−1^ using a solution of 2 U mL^−1^ thrombin and 0.3 U mL^−1^ aprotinin in complete vascular cell media. 10 µL of cell suspension was quickly mixed with 10 µL of 6 mg mL^−1^ fibrinogen, then 10 µL of this mixture (3 mg mL^−1^ fibrinogen, 1 U mL^−1^ thrombin, 0.15 U mL^−1^ aprotinin) was pipetted directly into the cell seeding port of the microfluidic device. Final total cell density and hydrogel formulation was based off the works of Andrée et al and Wan et al.^[^ ^25^, ^53^ ^]^ The cell‐seeded device was then placed in a humidified incubator for 15 min at 37 °C in 5% (v/v) CO_2_ in air to facilitate fibrin hydrogel formation. Media channels were then loaded with complete vascular cell media and reservoirs filled with ≈75 µL complete vascular cell media each. Devices were finally placed in a humidified incubator at 37 °C in 5% (v/v) CO_2_ in air with complete media exchanges every 2–3 days for up to 7‐days.

### Cell Culture and Imaging—*Time‐Lapse Imaging*

Time‐lapse microscopy of both 2D and 3D vasculogenesis was conducted using a Etaluma LS720 in‐incubator fluorescence microscope with automated stage control running Lumaview 720/600‐Series software. Images were acquired with minimum LED intensity to minimize phototoxicity and minimum photomultiplier gain to minimize image signal‐to‐noise. Acquisition was conducted across multiple wells of a 96‐well plate or the culture compartment of microfluidic devices using 4X and 10X objectives, respectively. Multipoint time‐lapse protocols were conducted with imaging intervals of 20–30 min over the course of 7‐days culture, capturing both FITC (excitation: 473–491 nm, emission: 502–561 nm) and brightfield channels.

### Cell Culture and Imaging—*3D Vasculogenesis‐Adipogenesis Gradient Co‐Culture*

Cell‐laden fibrin hydrogels were prepared in gradient microfluidic devices as per section 2.2.3. From days 0 to 3 of culture, one side of the device was loaded with complete vascular cell media and the other with complete MSC media, with full media exchanges on day‐1. On day‐3, complete vascular cell media was fully exchanged for fresh complete vascular cell media and complete MSC media with a half media exchange for adipocyte initiation media (PCS‐500‐050). This was replicated on day‐5. On day‐7, complete vascular cell media was fully exchanged for fresh complete vascular cell media and adipocyte initiation media, with a half media exchange for adipocyte maturation media (PCS‐500‐050). This was repeated every 2–3 days, up to day‐17. The culture gradient was then inverted on day‐17, whereby adipocyte maturation media was fully exchanged for fresh complete vascular cell media and complete vascular cell media with a half media exchange for adipocyte initiation media (PCS‐500‐050). The process from day‐3 to ‐17 was similarly repeated up to day‐31 with this inverted configuration. A schematic depicting this feeding strategy can be found in Figure 3.A. Cultures were additionally supplemented with 1 µM spermidine (0 5292) or 50 nM hydroxyl(oxo)vanadium 3‐hydroxypiridine‐2‐carboxylic acid (VO‐OHpic, 10 009 965) for the 31‐day duration.

### Cell Fixation, Immunofluorescence Staining and Imaging

Cultures were fixed at room temperature with 3.7% (w/v) paraformaldehyde (15 710) for 10 min then washed with DPBS three times for 5 min each. Fixed cultures were then permeabilized with 0.1% (w/v) Triton X‐100 solution in DPBS for 10 min followed by three 5‐min washes with DPBS, then incubated in 10% (v/v) normal goat serum (NGS, 50062Z) in DPBS blocking solution at 4 °C overnight. Cultures were then incubated in anti‐CD90 IgG (MA5‐32559), anti‐CD31 IgG2a (MA3100), anti‐aSMA IgG1 (MA5‐15806), anti‐PPARG IgG (MA5‐14889) and respective isotype control primary antibodies (31 235, 14‐4724‐82 and 14‐4724‐82), diluted as per Table S1 (Supporting Information) in 10% (v/v) NGS, for 1 h at room temperature, followed by three 5‐min washes with DPBS. Secondary antibodies anti‐rabbit IgG AlexaFluor488 (A‐11008), anti‐rat IgG AlexaFluor555 (A‐21434), anti‐mouse IgG2a AlexaFluor555 (A‐21137), anti‐rabbit IgG AlexaFluor647 (A‐21245), anti‐mouse IgG2a AlexaFluor647 (A‐21241) and anti‐mouse IgG1 AlexaFluor647 (A‐21240) were diluted to 4 µg mL^−1^ in 10% (v/v) NGS, or actin green solution (R37110) or actin red solution (R37112) diluted 2 drops mL^−1^ in DPBS, was incubated for 1 h at room temperature, followed by another three 5‐min washes with DPBS. 4′,6‐diamidino‐2‐phenylindole, dihydrochloride (DAPI) stock solution (R37606) diluted 2 drops mL^−1^ in DPBS was applied to cultures and incubated for 30 min at room temperature, followed by another three 5‐min washes with DPBS. For lipid staining, HCS LipidTox red neutral lipid stain (H34476) stock solution was diluted 1:200 in DPBS and applied to cultures overnight at 4 °C. Images were obtained using a Nikon Ti‐2 inverted fluorescence microscope or a Leica SP8 confocal laser scanning microscope. Confocal microscopy data was analyzed using Imaris 10.1.0 software.

### Culture Metabolic Activity Assay

Total culture metabolic activity was determined via PrestoBlue HS Cell Viability Reagent (P50200) as per manufacturers protocol. Briefly, a 1X working solution of PrestoBlue reagent was prepared by dilution of 10X stock solution in fresh room temperature culture media. Spent media was aspirated from cultures and replaced with PrestoBlue working solution and incubated for 30 min at 37 °C in 5% (v/v) CO_2_ in air. Following incubation, 90 µL samples of working solution was placed in Nunc F96 MicroWell Black Polystyrene Plates (237 105) and fluorescence intensity determined at an excitation wavelength of 560 nm and emission wavelength of 590 nm using an Ensight Multimode Micro Plate Reader (Perkin Elmer).

### Quantitative Image Analysis—*Vessel Network Morphology*

Fluorescence images of GFP‐hAECs were obtained as described in section 2.2.4 using a 4X objective with consistent illumination intensity, photomultiplier gain and exposure time. Images were captured with maximum vessel features in the plane of focus. Quantification of 2D and 3D vascular network morphology (including number of end points, number of junctions, total vessel length and total vessel area) was conducted using AngioTool version 0.6a,^[^ ^54^ ^]^ a lightweight open‐source software for quantitative analysis of vessel networks. Vessel network analysis was conducted at a user defined intensity scale which maximized accuracy of vessel network segmentation. Single cells and other fluorescent non‐vessel particles were excluded from the analysis using the software's “remove small particles” function and small holes and dark spots within vasculature were filled using the “fill holes” function.

### Quantitative Image Analysis—*Vessel Diameter Quantification*

Vessel diameters were measured in Fiji^[^ ^55^ ^]^ by manually drawing a line the thickness of a vessel approximately equidistant from two junctions. Pixel length was then converted into a distance measurement using a known scale reference.

### Quantitative Image Analysis—*Lipid Quantification*

Color or monochromatic images of 3D vasculogenesis‐adipogenesis gradient cultures were obtained at days‐17 and ‐31 of culture using an Olympus CKX 53 or Nikon Ti‐2 microscope. Images were captured at consistent illumination and gain values using the autoexposure camera function. Images were imported into ImageJ Fiji software and converted to 8‐bit format. Image threshold was adjusted to highlight dark lipid droplet‐like structures with spherical morphology in non‐vessel control cultures. These threshold parameters were used to analyses all other images. Regions of interest within the culture images were manually selected and percentage area of image within the threshold values determined.

### Growth Factor Distribution Modeling

Idealized growth factor distributions were simulated within the fibrin hydrogel culture compartment of the gradient microfluidic device (Figure 3). Following Altan‐Bonnet & Mukherjee,^[^ ^56^ ^]^ growth factors were assumed to degrade throughout culture via half‐life decay and transport from microfluidic channels into the hydrogel culture compartment via diffusion. In this simple model, growth factor media supplementation and half‐life decay were assumed to be significantly greater than other kinetics, such as growth factor secretion and cell or matrix sequestering, as found in the prior work modeling single‐cell cytokine release and binding.^[^ ^57^ ^]^ The composition of adipocyte initiation and maturation media is proprietary and therefore was approximated from the following source.^[^ ^58^ ^]^

Therefore, 2D growth factor transport and decay over time within the gradient microfluidic device were simply modeled as:

(1) $$\frac{dC}{dt}=D\left(\frac{\partial^{2}C}{\partial x^{2}}+\frac{\partial^{2}C}{\partial y^{2}}\right)+kC$$

where, growth factor concentration *C* is modelled as a partial differential equation of dimensions *x*, *y* and *t*. Here, *D* represents a growth factor's diffusivity constant (µm^2^ s^−1^) and half‐life decay rate *k* is equal to *ln(0.5/t_1/2_)* with *t_1/2_* being the time for the growth factor to decay to half its original concentration. It is important to note this simulation also applies the same half‐life decay to the microchannel boundary concentration conditions, as media is only replenished every 2–3 days.

The microchip's 3D fibrin hydrogel cell culture chamber is 1.3 mm wide and 7.9 mm long, where four media channels interface with the culture chamber at its top‐left, top‐right, bottom‐left, and bottom‐right corners for 2.6 mm of length, each. **Table** **1** lists major growth factors’ initial conditions, diffusivity, half‐life, half maximal effective concentration (EC50) from these experiments. It is important to note that these parameters were derived from 2D culture conditions and growth factor transport, decay and effect would likely change in hydrogels.^[^ ^59^ ^]^ In addition, the diffusion and half‐life kinetics of adipogenic factor IBMX (3‐isobutyl‐1‐methylxanthine) were unable to be identified and modeled.

**Table 1:** Growth factor distribution modelling parameters. Blue, red, and yellow rows represent critical stromal, vascular, and adipogenic growth factors, respectively. It is important to note that EGF and FGF‐β are included in both stromal (MSC) and vascular (EC) media. IGF‐1: insulin‐like growth factor 1, VEGF: vascular endothelial growth factor, EGF: epidermal growth factor, FGF‐β: fibroblast growth factor‐basic, FGF‐α: fibroblast growth factor‐acidic, DEX: dexamethasone.

	Growth Factor and References	Supplemented Concentration [ng mL^−1^]	Molecular Weight [g mol^−1^]	Diffusivity [µm^2^ s^−1^]	Half‐life [s]	Half‐effective concentration [EC50; ng mL^−1^]
**Stromal**	FGF‐α^[^ ^61^, ^62^, ^63^, ^64^, ^65^ ^]^	5	15800	100	4320	0.3
	EGF^[^ ^66^, ^67^, ^68^, ^69^, ^70^, ^71^ ^]^	5	6400	21	25200	0.1
	FGF‐β^[^ ^72^, ^73^, ^74^ ^]^	5	17100	92	90000	0.1
**Vascular**	IGF‐1^[^ ^75^, ^76^ ^]^	15	7600	159	1440	5
	VEGF^[^ ^77^, ^78^, ^79^, ^80^ ^]^	5	38200	200	3600	1.7
**Adipogenic**	DEX^[^ ^81^, ^82^ ^]^	392	392	10.8	16200	1.9
	Insulin^[^ ^83^, ^84^, ^85^ ^]^	1000	5808	200	108000	33.7

The distribution of each growth factor was individually simulated across the microfluidic cell culture compartment in Figure S18 (Supporting Information). FGF‐α, IGF‐1, and DEX were respectively identified to be the limiting growth factors for stromal, vasculogenic, or adipogenic media distribution. That is, these growth factors were predicted to distribute the shortest distance across microfluidic chip culture, while still maintaining bioactive concentrations. A short growth factor distribution could be due to smaller diffusivity, shorter half‐life, or lower supplemented concentration relative to higher half‐effective concentrations (EC50). Detailed simulations of these limiting growth factors (FGF‐α, IGF‐1, DEX) were performed in Figure 3. The Python simulation code is provided in the supplementary information for an exemplary pair of counter‐current growth factors, where its approach in implementing a 2D Laplacian for growth factor diffusion follows the methodology of Rossant.^[^ ^60^ ^]^

### Statistical Analysis

Unless otherwise stated in figure captions, numerical results are reported as the mean average of biological replicates ± standard deviation. Unless otherwise stated in the Figure caption or text, all experiments include three biological replicates, each with three technical replicates. Statistical analysis includes ordinary one‐way ANOVA with a Tukey multiple comparisons test (α = 0.05) and ordinary two‐way ANOVA (α = 0.05) with a Tukey multiple comparisons test (α = 0.05). *p* value output style is *p* > 0.05 (ns), *p* ≤ 0.05 (*), *p* ≤ 0.01 (**), *p* ≤ 0.001 (***) and *p* ≤ 0.0001 (****). All statistical analysis and multiple comparison tests were performed with Graph Pad Prism 10.3.0 for Windows (GraphPad Software, Boston, Massachusetts USA, www.graphpad.com), and are detailed in Figure captions.

## Conflict of Interest

The authors declare no conflict of interest.

## Supporting information



Supporting Information

Supplemental Video 1

Supplemental Video 2

Supplemental Video 3

## Data Availability

The data that support the findings of this study are available from the corresponding author upon reasonable request.
