# Supplementary material for: Fabricating Microfluidic Co‐Cultures of Immortalized Cell Lines Uncovers Robust Design Principles for the Simultaneous Formation of Patterned, Vascularized, and Stem Cell‐Derived Adipose Tissue
Source: Small. 2025 Jun 17;21(32):2501834. doi: 10.1002/smll.202501834 (PMC12366273; doi:10.1002/smll.202501834)
Supplement: Supplementary file 1 — Supporting Information [file SMLL-21-2501834-s002.docx]

| Supplier | Catalogue Number | Antigen | Species Reactivity | Host, Isotype | Stock Concentration (mg/mL) | Working Concentration (µg/mL) |
| --- | --- | --- | --- | --- | --- | --- |
| ThermoFisher Scientific | MA5-32559 | CD90 | Human | Rabbit, IgG | 1 | 10 |
| ThermoFisher Scientific | MA3100 | CD31 | Human | Mouse, IgG2a | 1 | 10 |
| ThermoFisher Scientific | MA5-15806 | aSMA | Human | Mouse, IgG1 | Not specified | (1:250 dilution) |
| ThermoFisher Scientific | MA5-14889 | ppARg | Human | Rabbit, IgG | 0.125 | 1.25 |
| ThermoFisher Scientific | 14-5698-82 | Ki67 | Human | Rat, IgG2a | 0.5 | 5 |
| ThermoFisher Scientific | 31235 | IgG isotype control | - | Rabbit, IgG | 11 | 10, 1.25 |
| ThermoFisher Scientific | 14-4724-82 | IgG2a isotype control | - | Mouse, IgG2a, kappa | 0.5 | 10 |
| ThermoFisher Scientific | 14-4714-82 | IgG1 isotype control | - | Mouse IgG1, kappa | 0.5 | (1:250 dilution) |
| ThermoFisher Scientific | A-11008 | Goat anti-Rabbit IgG (H+L), Alexa Fluor™ 488 | Rabbit | Goat, IgG | 2 | 4 |
| ThermoFisher Scientific | A-21434 | Goat anti-Rat IgG (H+L), Alexa Fluor™ 555 | Rat | Goat, IgG | 2 | 4 |
| ThermoFisher Scientific | A-21137 | Goat anti-Mouse IgG2a, Alexa Fluor™ 555 | Mouse | Goat, IgG | 2 | 4 |
| ThermoFisher Scientific | A-21245 | Goat anti-Rabbit IgG (H+L), Alexa Fluor™ 647 | Rabbit | Goat, IgG | 2 | 4 |
| ThermoFisher Scientific | A-21241 | Goat anti-Mouse IgG2a, Alexa Fluor™ 647 | Mouse | Goat, IgG | 2 | 4 |
| ThermoFisher Scientific | A-21240 | Goat anti-Mouse IgG1, Alexa Fluor™ 647 | Mouse | Goat, IgG | 2 | 4 |

**Table S1.** Primary and secondary antibody details


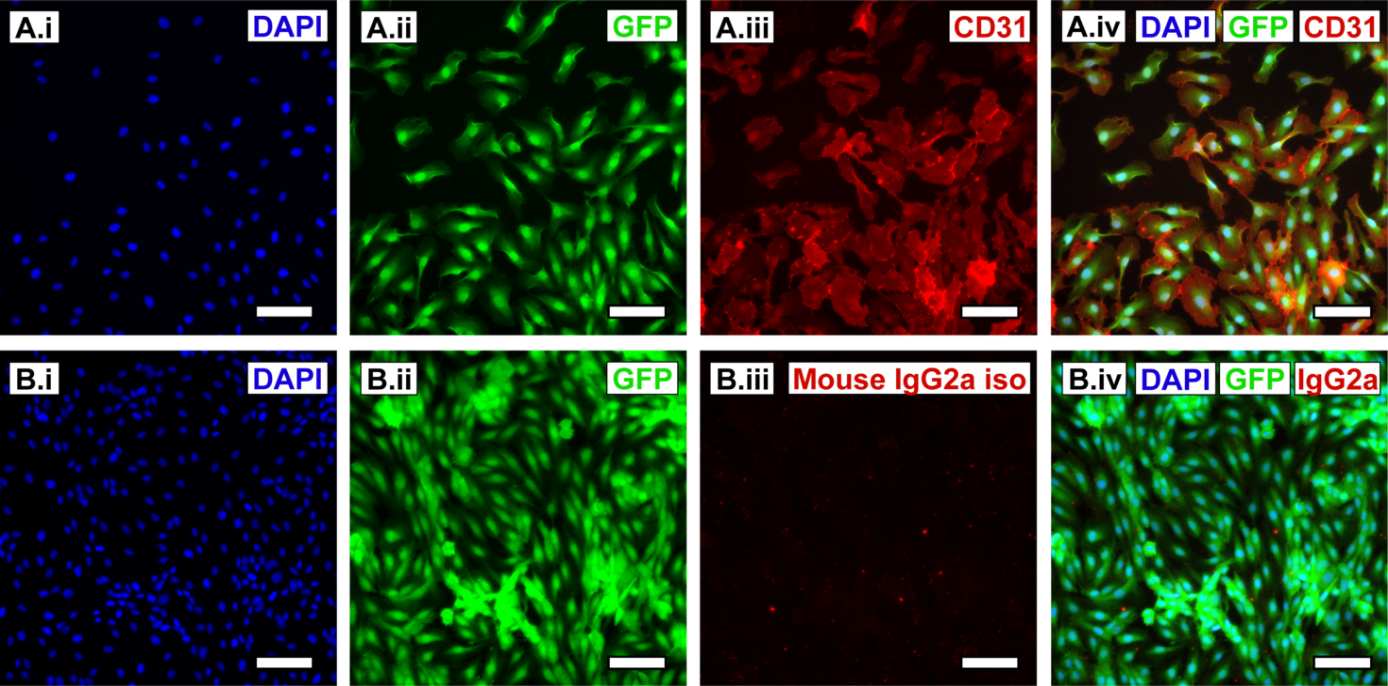


**Figure S1. GFP-hAEC immunocytochemical (ICC) fluorescence characterisation. A.** ICC detection for cluster of differentiation 31 (CD31) with images representing nuclear counterstain DAPI (**i**), endogenous GFP expression (**ii**), CD31 (**iii**) and a merge of the three (**iv**). **B.** ICC detection for the mouse IgG2a isotype control antibody with images representing nuclear counterstain DAPI (**i**), endogenous GFP expression (**ii**), mouse IgG2a isotype control (**iii**) and a merge of the three (**iv**). Scale bars = 100 µm. DAPI: 4’,6-diamidino-2-phenylindole, GFP: green fluorescent protein, CD31: cluster of differentiation 31, iso: isotype, M: mouse, IgG: immunoglobulin G.


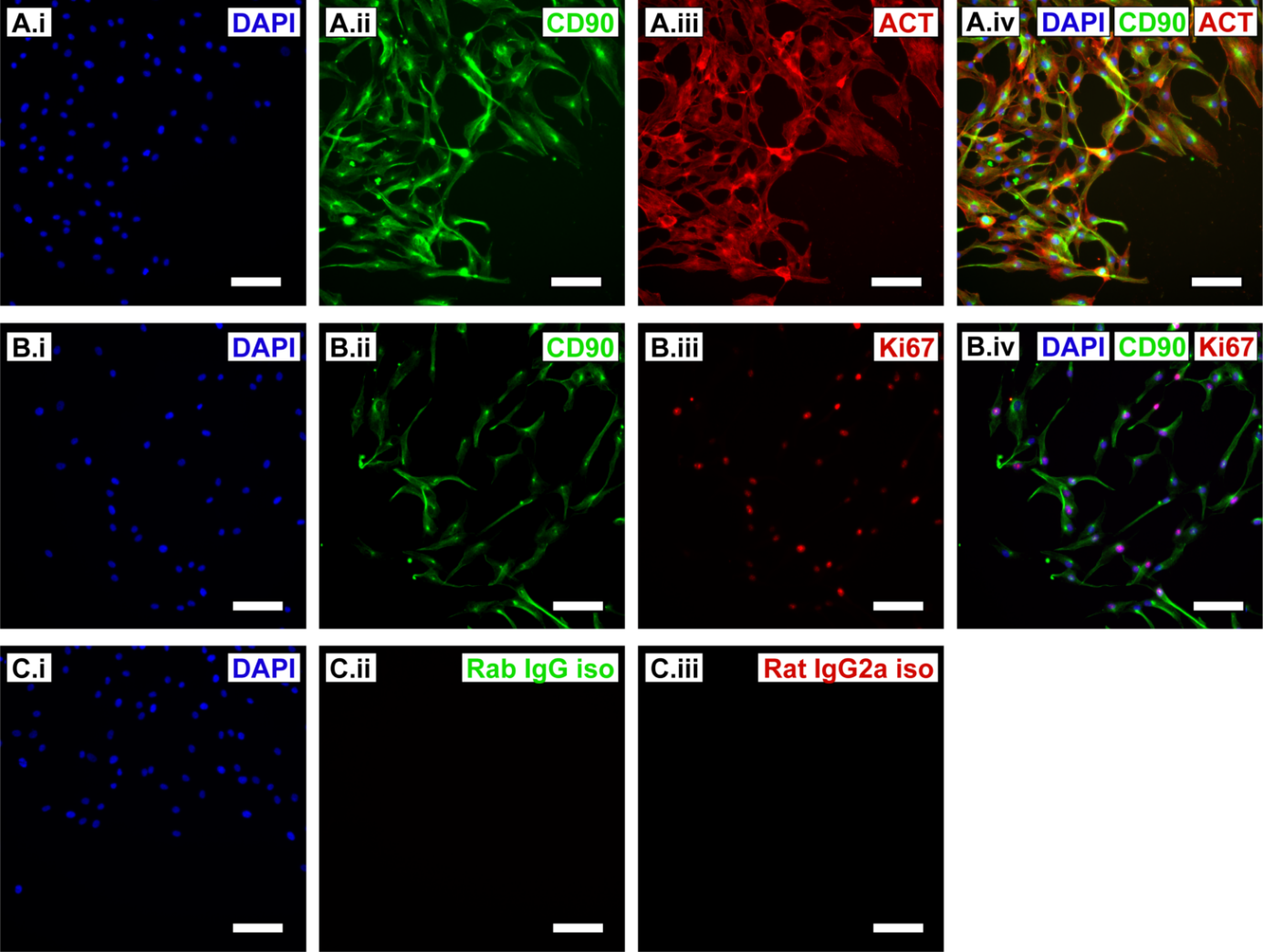


**Figure S2. hAD-MSC immunocytochemical (ICC) fluorescence characterisation. A.** ICC detection for cluster of differentiation 90 (CD90) with images representing nuclear counterstain DAPI (**i**, blue), CD90 (**ii**, green), filamentous actin (**iii**, red) and a merge of the three (**iv**). **B.** ICC detection for Kiel 76 (Ki67) with images representing nuclear counterstain DAPI (**i**, blue), CD90 (**ii**, green), Ki67 (**iii**, red) and a merge of the three (**iv**). **C.** ICC detection for isotype control antibodies with images representing nuclear counterstain DAPI (**i**, blue), rabbit IgG isotype (**ii**, green) and rat IgG2a isotype (**iii**, red). Scale bars = 100 µm. DAPI: 4’,6-diamidino-2-phenylindole, CD90: cluster of differentiation 90, IgG: immunoglobulin G, iso: isotype, rab: rabbit.

**Video S1. Time-lapse microscopy of GFP-hAECs and hAD-MSCs captures spontaneous formation of 2D microvascular networks with varying seeding ratio.** Fluorescence time-lapse imaging (20 minute intervals) of GFP-hAEC microvascular networks formed over 7-days culture in complete vascular cell media, with GFP-hAEC:hAD-MSC seeding ratios of 2:1 , 1:1, 1:2, 1:5 and 1:10 (scale bars = 1 mm).


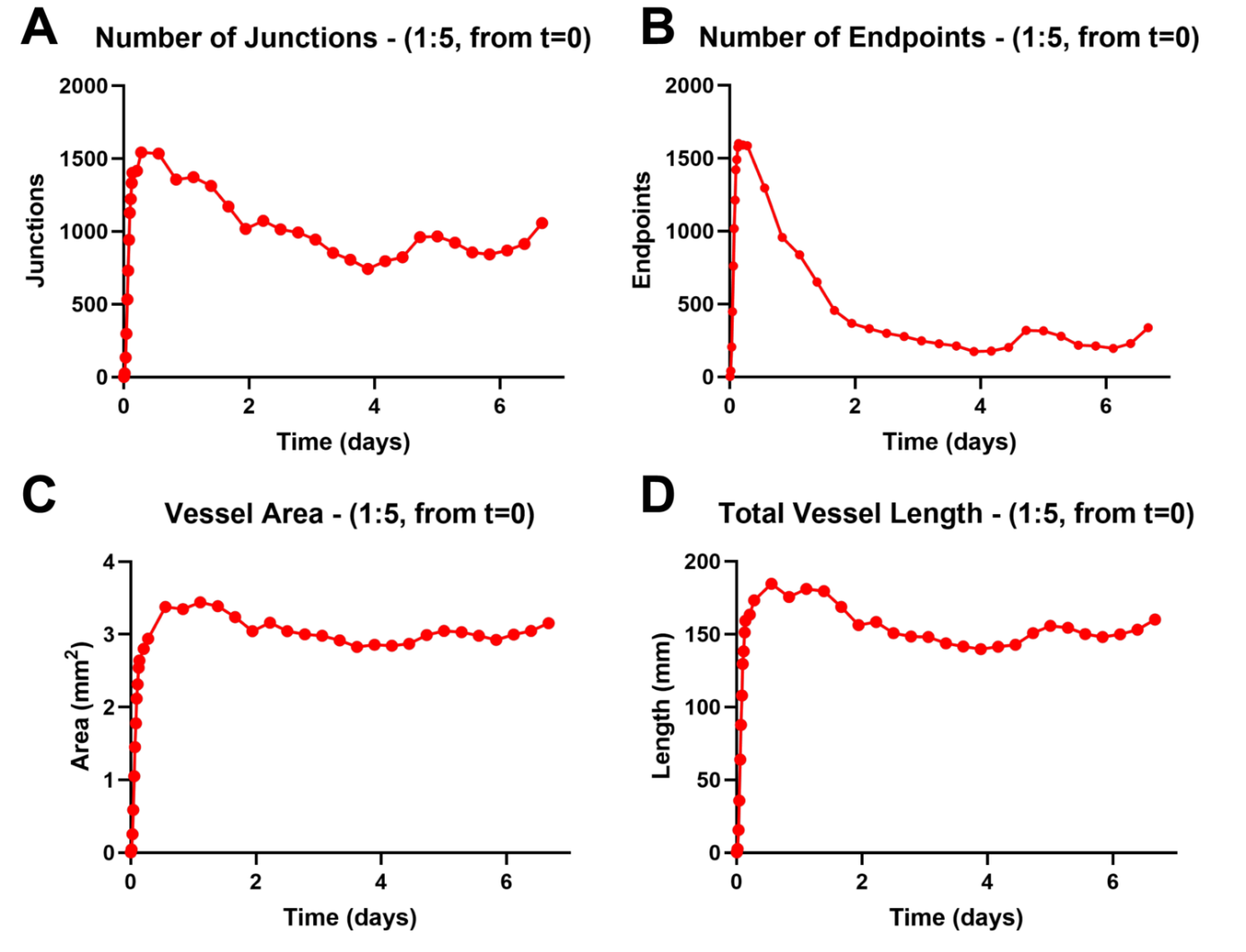
**Figure S3. GFP-hAEC and hAD-MSC vasculogenesis co-culture time-lapse quantification.** Quantitative morphological analysis of image set commenced directly post-seeding for the entire length of the culture. **A.** Number of vessel network junctions. **B.** Number of vessel endpoints. **C.** Total vessel network area. **D.** Total vessel network length.


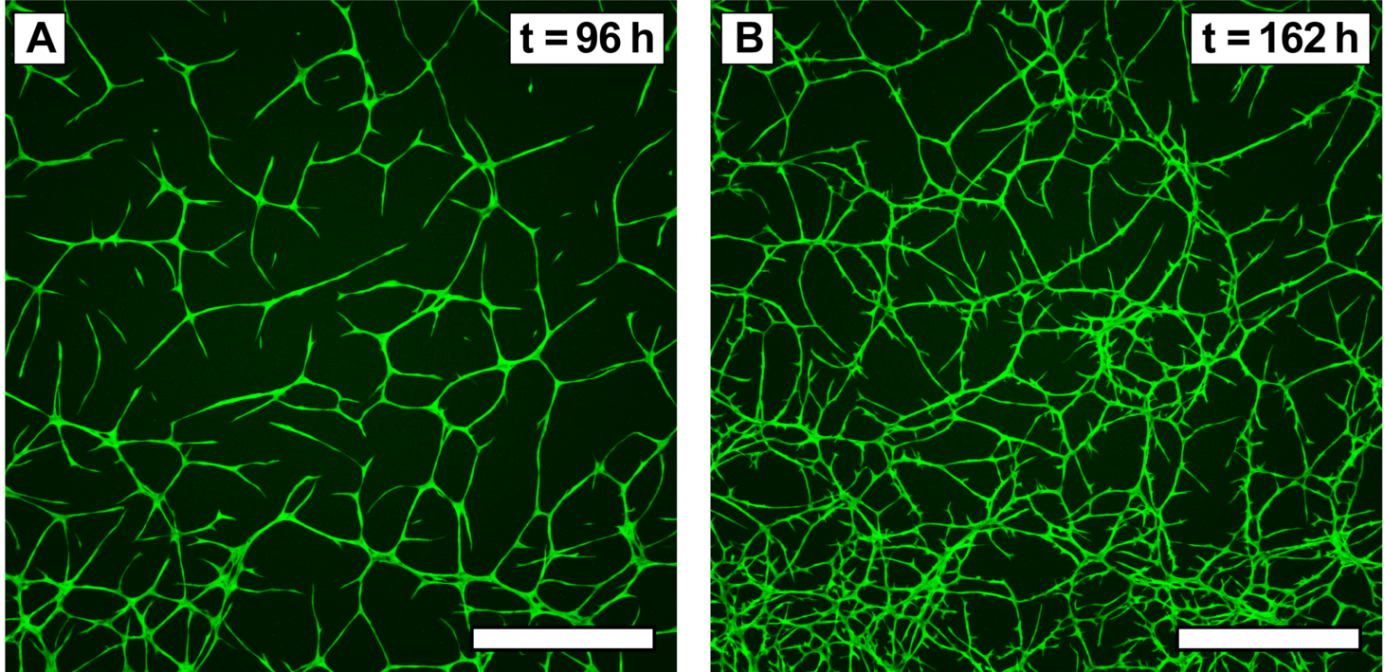


**Figure S4. GFP-hAEC and hAD-MSC vasculogenesis 1:10 co-cultures exhibit angiogenesis-like behaviour in the latter stages of a 7-day culture. A.** At the 96^th^ hour of culture, vessels display elongated morphology with no spine (scale bar = 1 mm). **B.** At the 162^nd^ hour of culture, vessels develop short spines extending from long continuous vessels (scale bar = 1 mm).


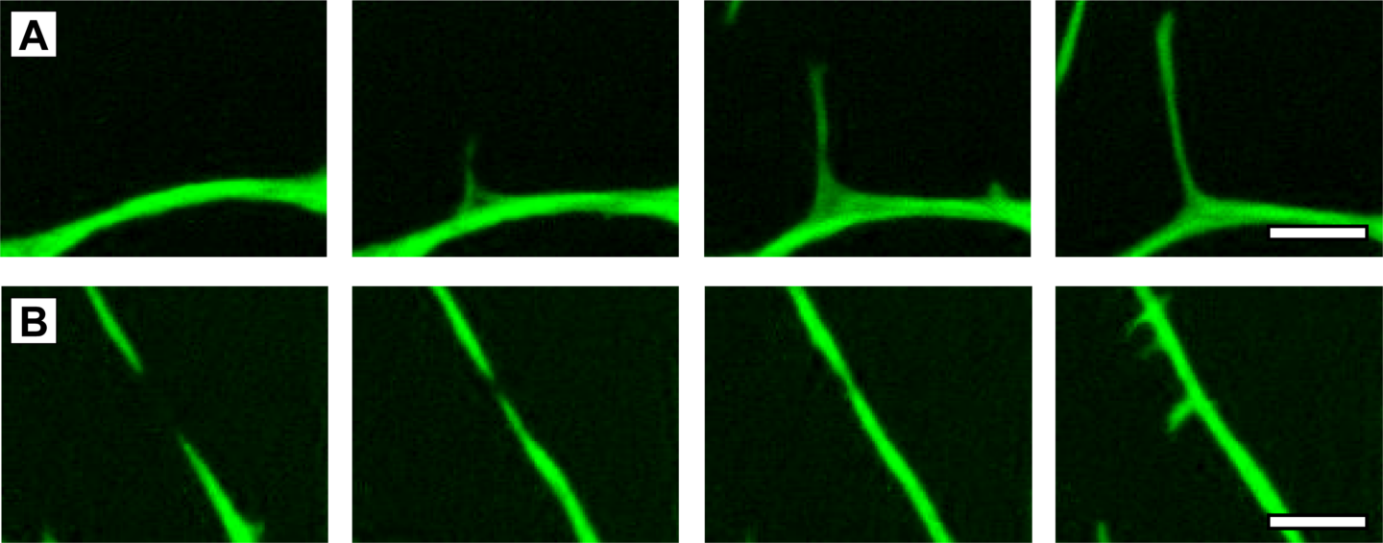


**Figure S5. Vasculogenesis cultures replicate vessel morphogenetic processes other than vasculogenesis. A.** Angiogenesis can be observed as the protrusion of a single tip from an existing vessel which grows in length over time (scale bar = 100 µm). **B.** End-to-end anastomosis can also be observed when two vessel tips grow towards each other and eventually fuse into a single continuous vessel (scale bar = 100 µm).


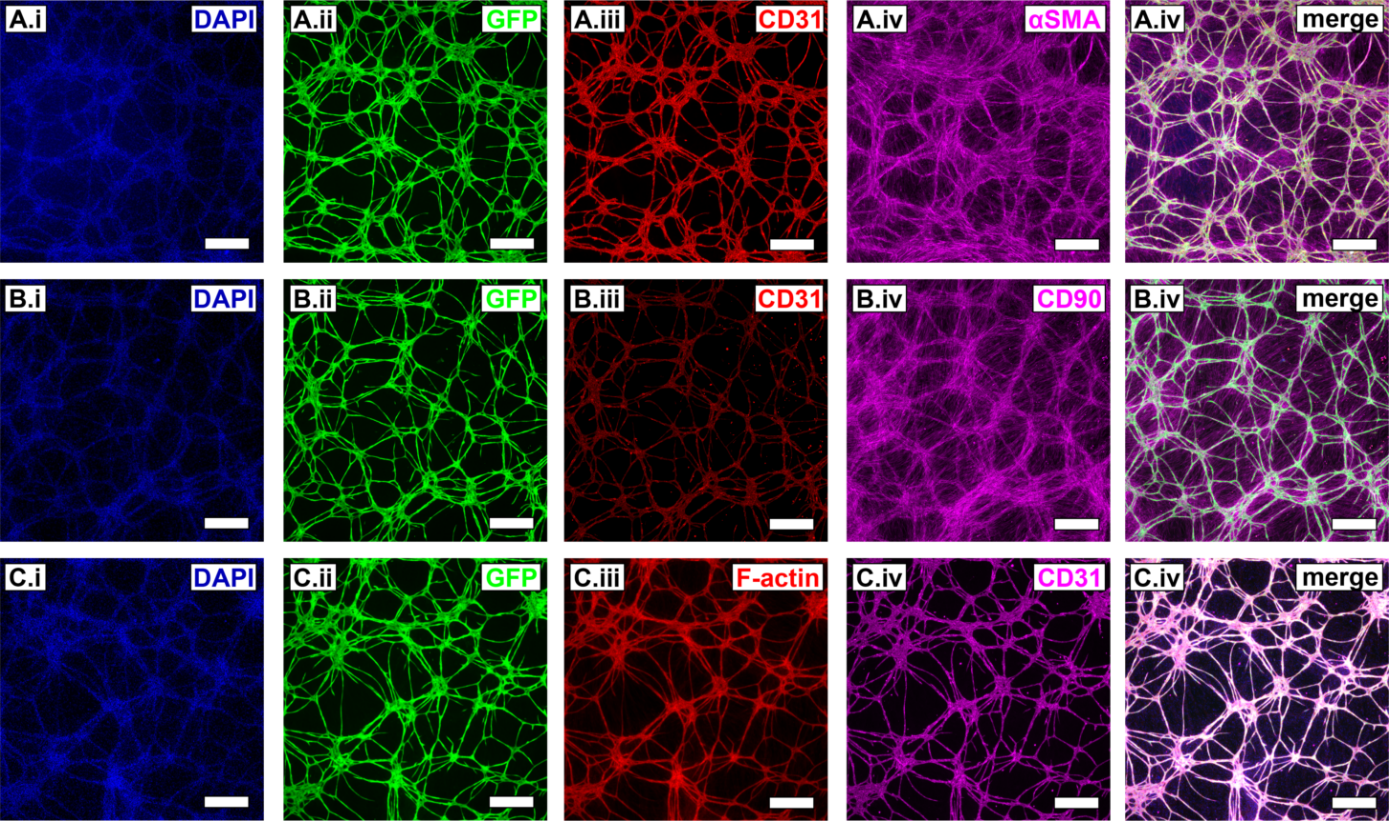


**Figure S6. GFP-hAEC and hAD-MSC vasculogenesis co-culture day 7 (seeded at 1:2 ratio) immunocytochemical (ICC) fluorescence characterisation. A.** Images representing nuclear counterstain DAPI (**i**, blue), GFP (**ii**, green), CD31 (**iii**, red), αSMA (**iv**, magenta) and a merge of the four (**v**). **B.** Images representing nuclear counterstain DAPI (**i**, blue), GFP (**ii**, green), CD31 (**iii**, red), CD90 (**iv**, magenta) and a merge of the four (**v**). **C.** Images representing nuclear counterstain DAPI (**i**, blue), GFP (**ii**, green), filamentous actin (**iii**, red), CD31 (**iv**, magenta) and a merge of the four (**v**). Scale bars = 500 µm. DAPI: 4’,6-diamidino-2-phenylindole, GFP: green fluorescent protein. DAPI: 4′,6-diamidino-2-phenylindole, GFP: green fluorescent protein, CD31: cluster of differentiation 31, αSMA: alpha smooth muscle actin, CD90: cluster of differentiation 90, F-actin: filamentous actin.


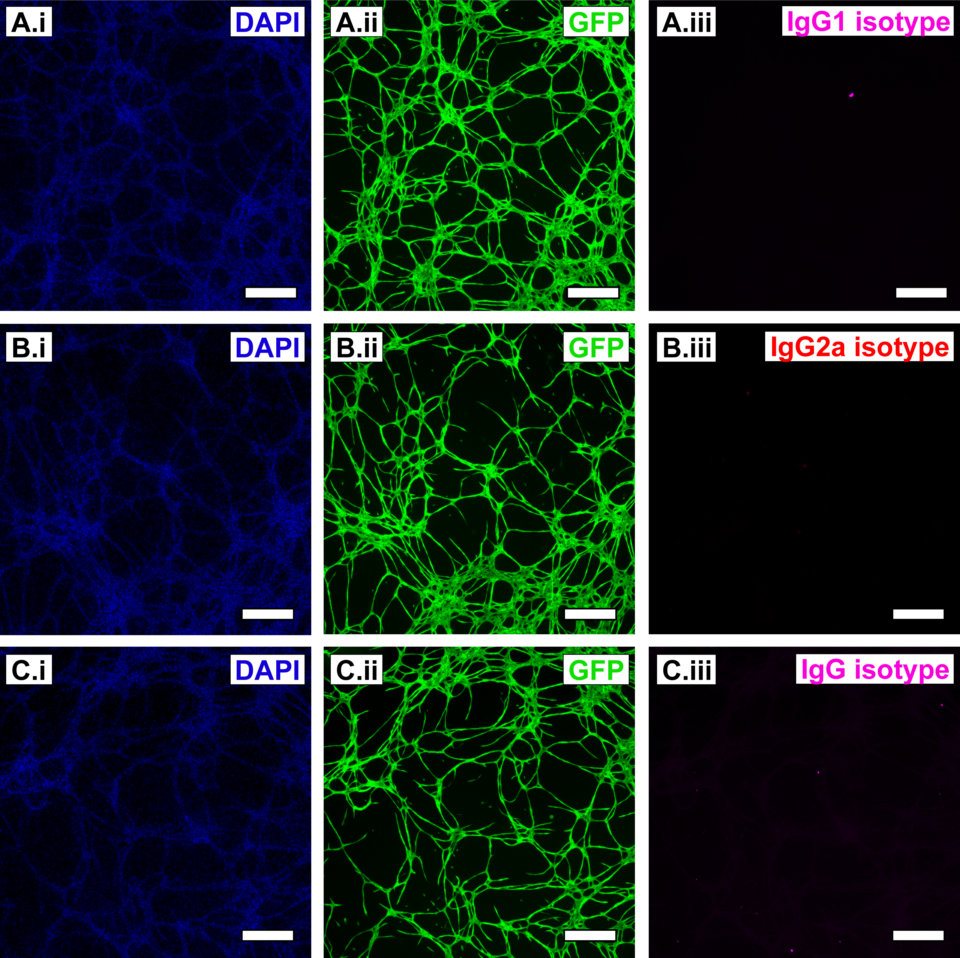


**Figure S7. GFP-hAEC and hAD-MSC vasculogenesis co-culture day 7 (seeded at 1:2 ratio) immunocytochemical (ICC) fluorescence characterisation, isotype control stains. A.** Mouse IgG1 isotype control with images representing nuclear counterstain DAPI (**i**, blue), GFP (**ii**, green), mouse IgG isotype control antibody (**iii**, magenta). **B.** Mouse IgG2a isotype control with images representing nuclear counterstain DAPI (**i**, blue), GFP (**ii**, green), mouse IgG2a isotype control antibody (**iii**, red). **C.** Rabbit IgG isotype control with images representing nuclear counterstain DAPI (**i**, blue), GFP (**ii**, green), rabbit IgG isotype control antibody (**iii**, magenta). Scale bars = 500 µm. DAPI: 4’,6-diamidino-2-phenylindole, GFP: green fluorescent protein.


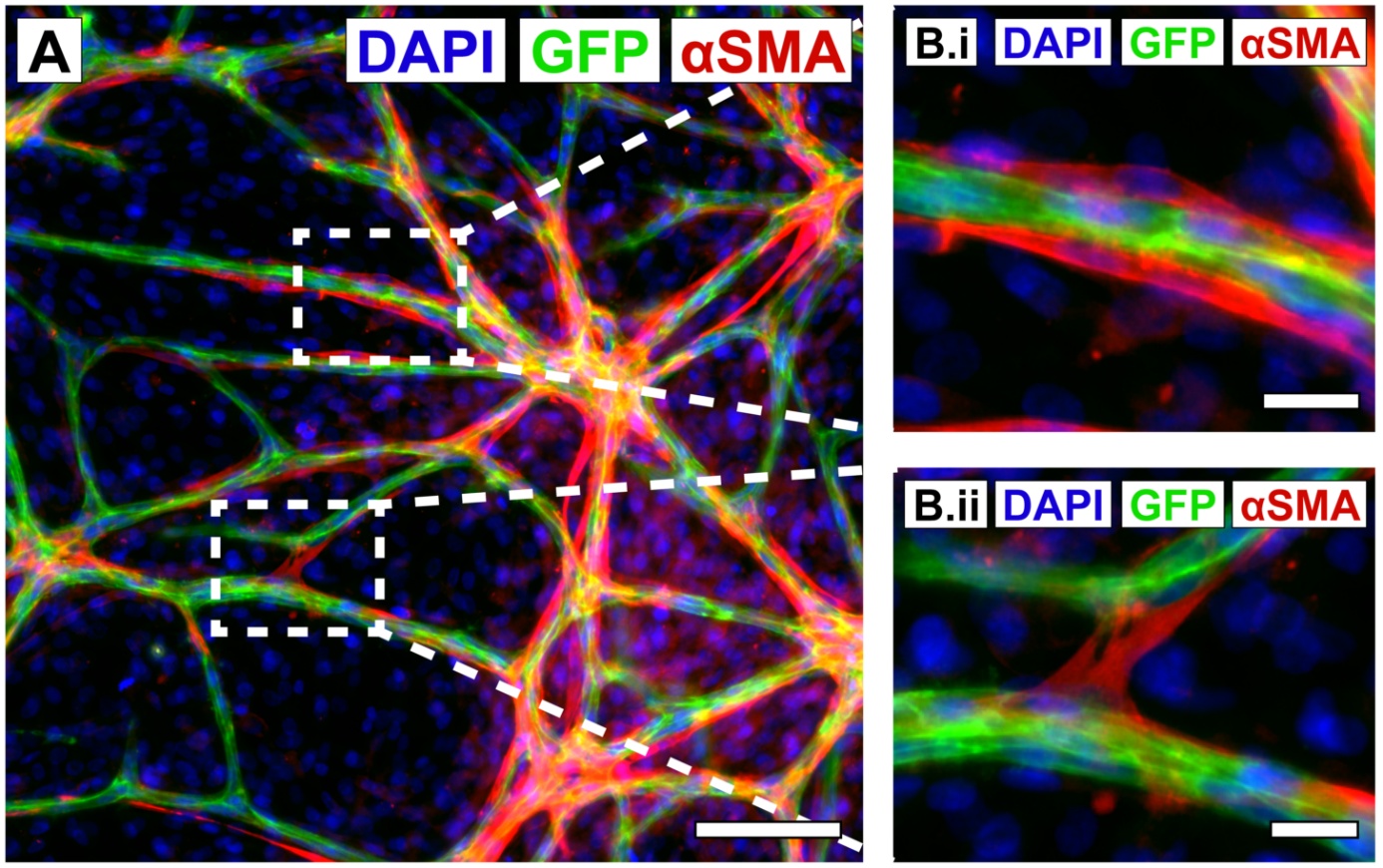


**Figure S8. GFP-hAEC and hAD-MSC vasculogenesis co-cultures display pericyte-like behaviour. A.** Vasculogenesis 1:5 cultures fixed at day 7 and immunocytochemically stained for alpha smooth muscle actin (αSMA), nuclear counter stained with DAPI and captured for endogenous GFP (scale bar = 100 µm). **B.** Cells positive for αSMA are observed lining the outside of vessels (i, scale bar = 20 µm) and forming bridges between vessels (ii, scale bar = 20 µm).


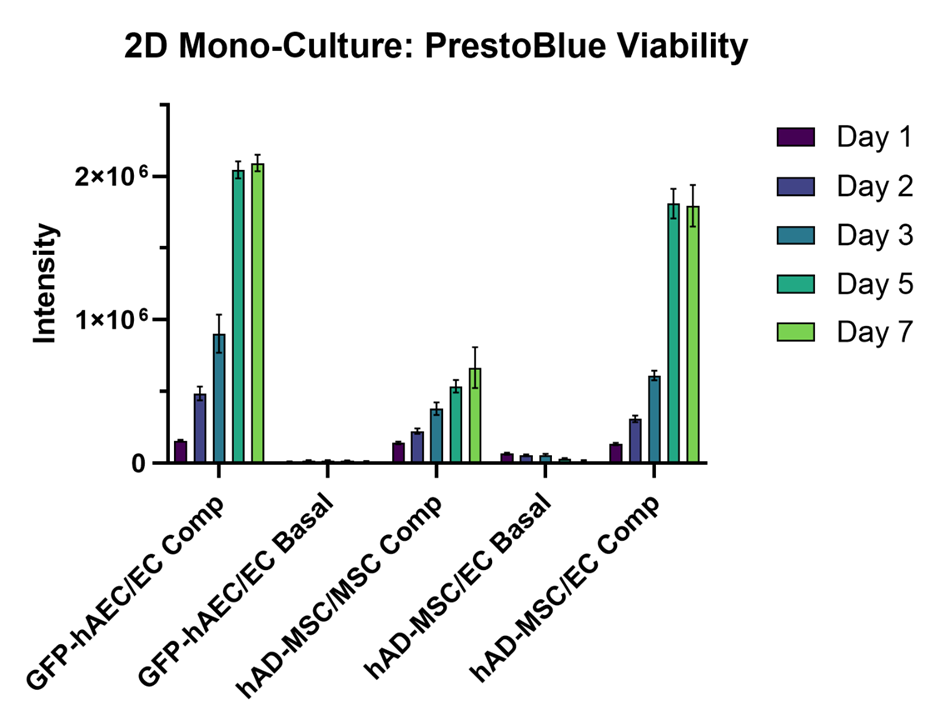


**Figure S9. GFP-hAECs and hAD-MSCs demonstrate altered culture viability in the presence of different media compositions.** GFP-hAECs were culture in complete vascular cell media and vascular cell basal cell media; and hAD-MSCs in complete MSC media, MSC basal media and complete vascular cell media. EC comp: complete vascular cell media, EC basal: vascular cell basal media, MSC comp: complete MSC media. (n = 3 technical replicas)


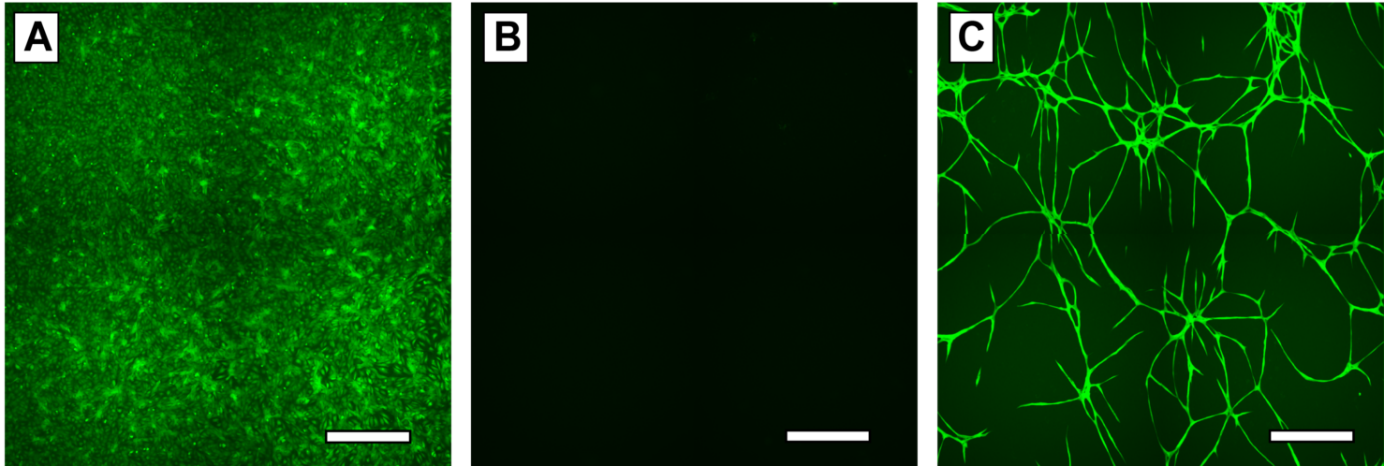


**Figure S10. In 2D culture conditions, GFP GFP-hAECs only form vessel networks in combination with endothelial cell growth factors and supporting hAD-MSCs. A.** In the absence of hAS-MSCs, GFP-hAECs form a dense monolayer after 7-days culture (scale bar = 500 µm). **B.** In the absence of vascular cell growth factors, GFP-hAECs do not attach to the culture surface (scale bar = 500 µm). C. In the presence of hAD-MSCs and complete vascular cell media, GFP-hAECs spontaneously form vessel networks after 7-days culture (scale bars = 500 µm).


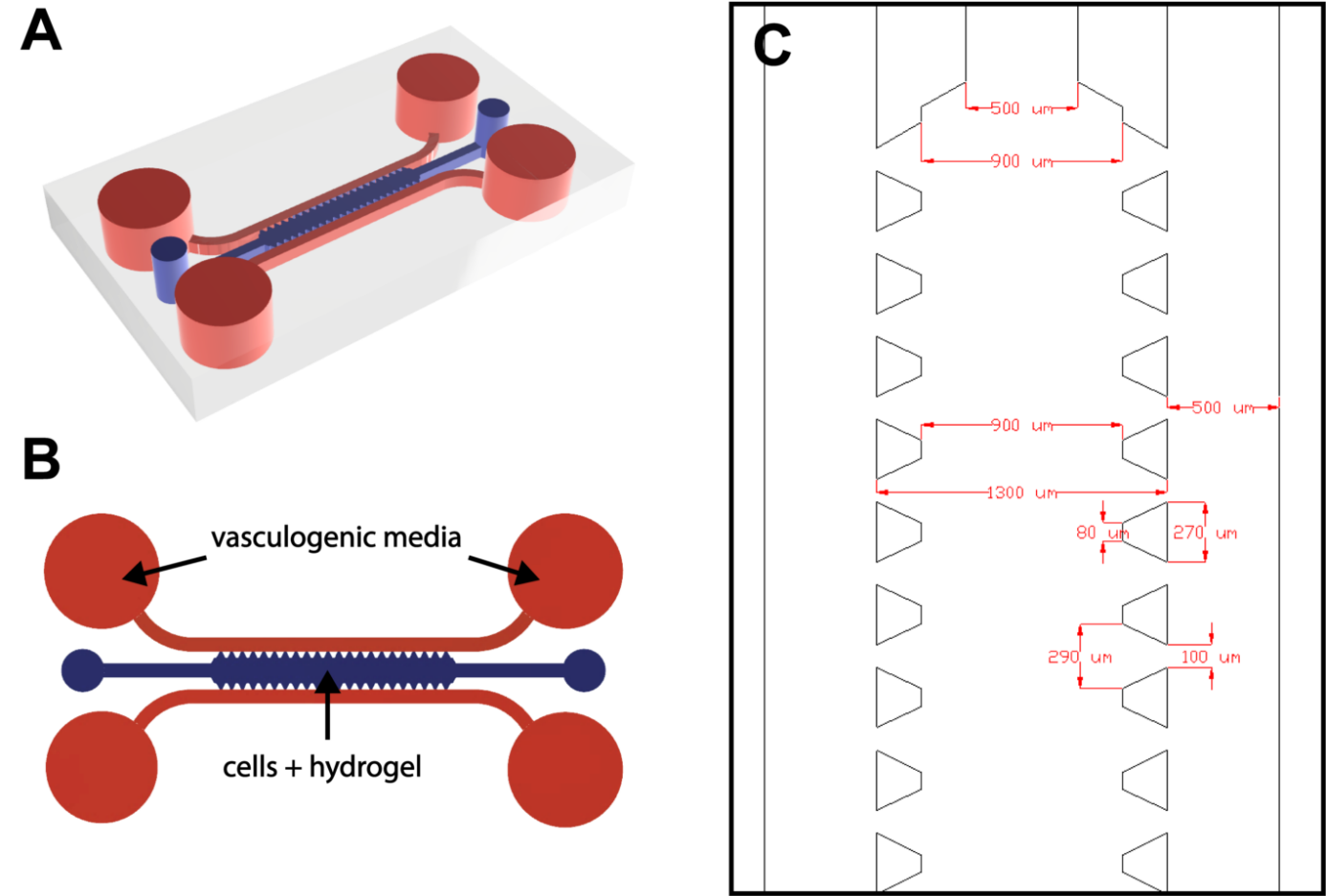


**Figure S11. Microfluidic culture device for supporting 3D hydrogel embedded vasculogenesis. A.** Device rendering. **B.** Device schematic depicting the cell-hydrogel culture compartment (blue) and flanking vasculogenic media channels (red). **C.** Device dimensions.

**Video S2. Time-lapse microscopy GFP-hAECs and hAD-MSCs captures spontaneous formation of 3D microvascular networks.** Fluorescence (left) and brightfield (right) time-lapse imaging (30 minute intervals) of GFP-hAEC microvascular networks formed over 7-days culture in complete vascular cell media, with GFP-hAEC:hAD-MSC seeding ratios of 1:1 (field of view = 1.33 × 1.33 mm).

**Video S3. Time-lapse microscopy and segmented image analysis outputs of GFP-hAECs and hAD-MSCs captures and quantified spontaneous formation of 3D microvascular networks.** Fluorescence (left) and overlayed segmentation and skeletonization (right) of time-lapse imaging (300 minute intervals) of GFP-hAEC microvascular networks formed over 7-days culture in complete vascular cell media, with GFP-hAEC:hAD-MSC seeding ratios of 1:1 (field of view = 1.33 × 1.33 mm).


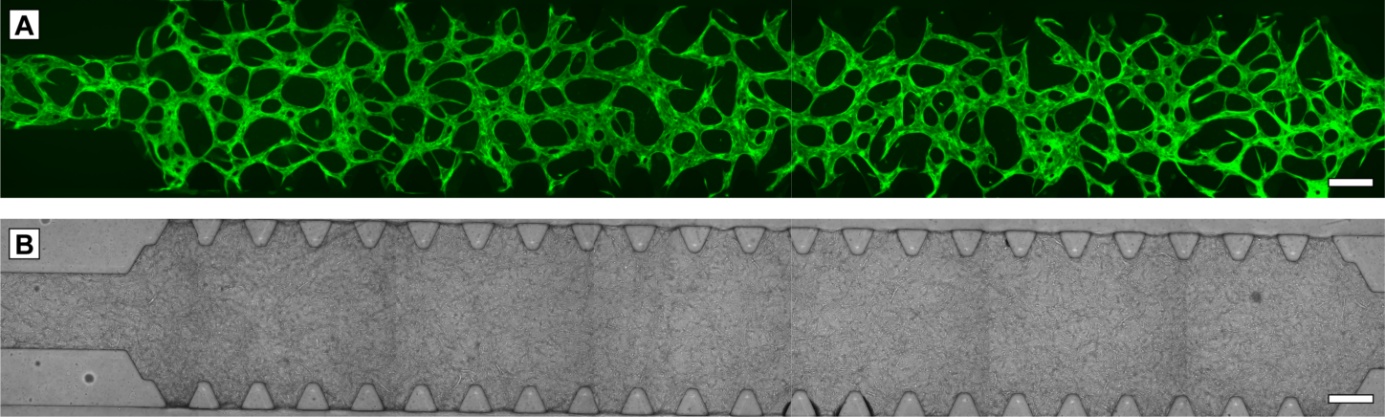


**Figure S12. GFP-hAECs and hAD-MSCs spontaneously form robust 3D hydrogel-embedded microvascular networks across the entire length of microfluidic device culture compartments.** Fluorescence (*A*) and Brightfield (*B*) images of GFP-hAECs as microvascular networks formed after 7-days fibrin hydrogel-embedded microfluidic device culture in complete vascular cell media with GFP-hAEC:hAD-MSC seeding ratios of 1:1 (scale bars = 300 µm).


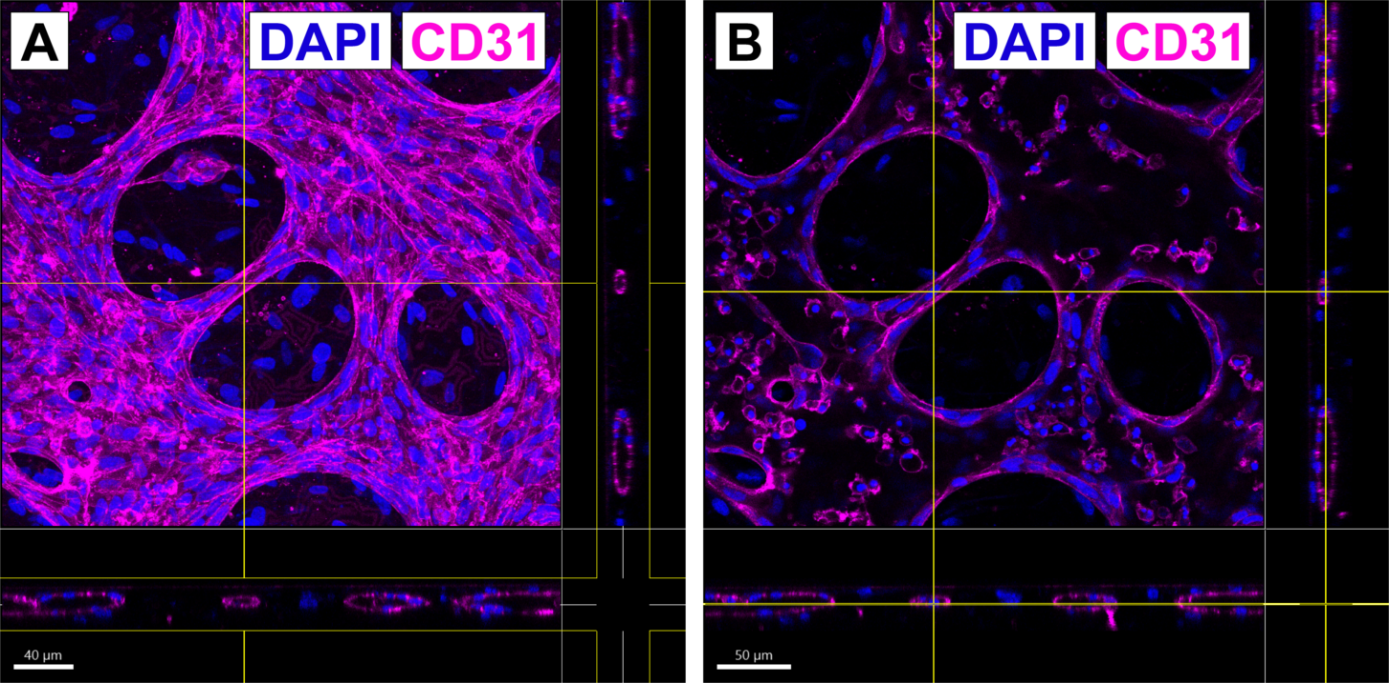


**Figure S13. Single CD31 positive cells reside within microvascular network lumen. A.** Maximum intensity projection of a CD31^+^ vessel network demonstrated vessel patency in the *xz* and *yz* cross-sectional plane (scale bar = 40 µm). B. Single round CD31^+^ cells can be seen suspended in the *xy* plane bisecting the vessel network (scale bar = 50 µm). DAPI: 4’,6-diamidino-2-phenylindole, CD31: cluster of differentiation 31.


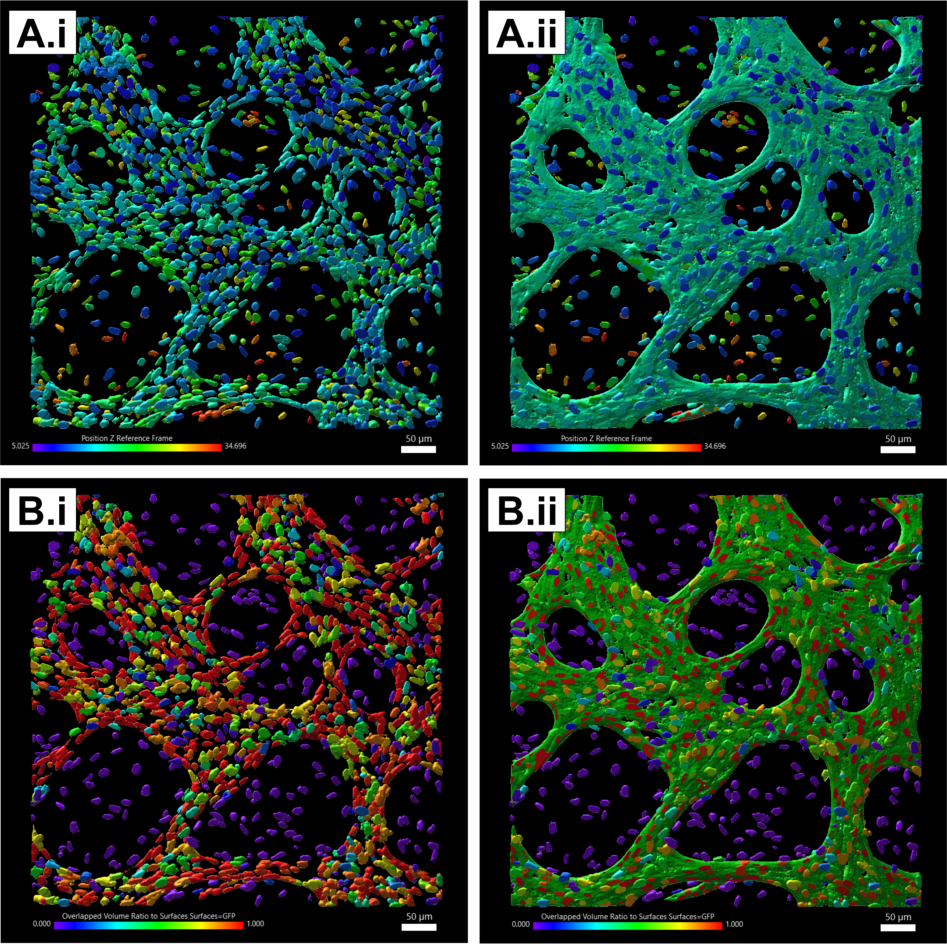


**Figure S14. Cell nuclei occupy extra-vascular space between, above and below microvascular networks. A.** Z-position depth map depicting the height (µm) of segmented cell nuclei within the 582 × 582 × 35.6 µm field of view displayed in the absence (i) and presence of segmented microvascular network GFP (scale bars = 50 µm). **B.** Overlap map depicting the percentage overlap of a cell nuclei with the segmented microvascular network GFP, displayed in the absence (i) and presence of segmented microvascular network GFP (scale bars = 50 µm).
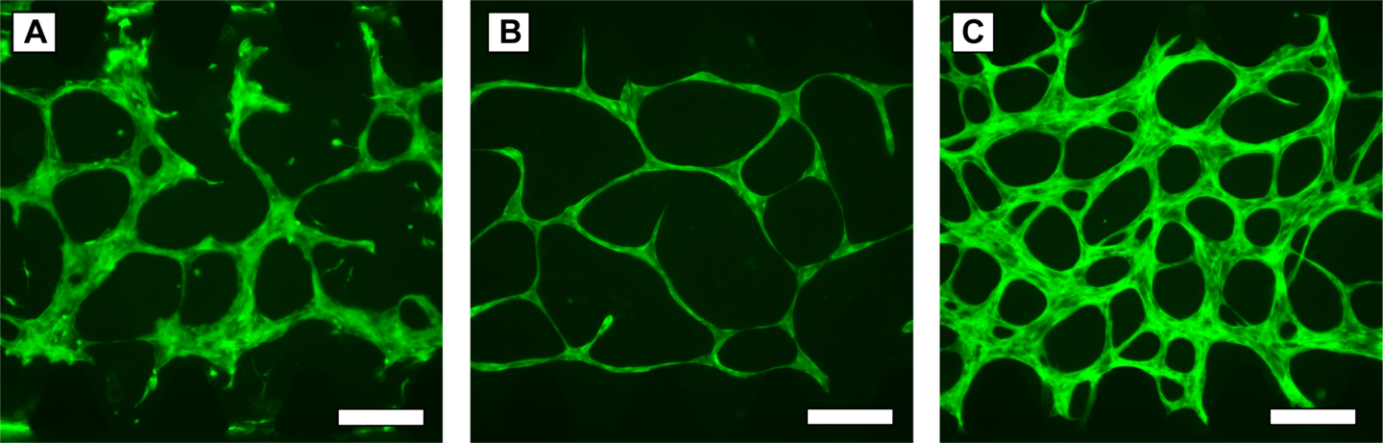


**Figure S15. Vessel networks form in altered culture conditions with variable quality. A.** In the absence of adipose-derived mesenchymal stem cells (hAD-MSCs) vessel networks form with discontinuities and rough borders (scale bar = 300 µm). **B.** In the absence of vascular cell growth factors (in the presence of hAD-MSCs, 1:1) continuous vessel networks with tight borders and abnormally thin vessel diameters form (scale bar = 300 µm). **C.** In the presence of both hAD-MSCs (1:1 seeding ratio) and vascular cell growth factors continuous vessel networks with tight borders and typical vessel diameters form (scale bar = 300 µm).


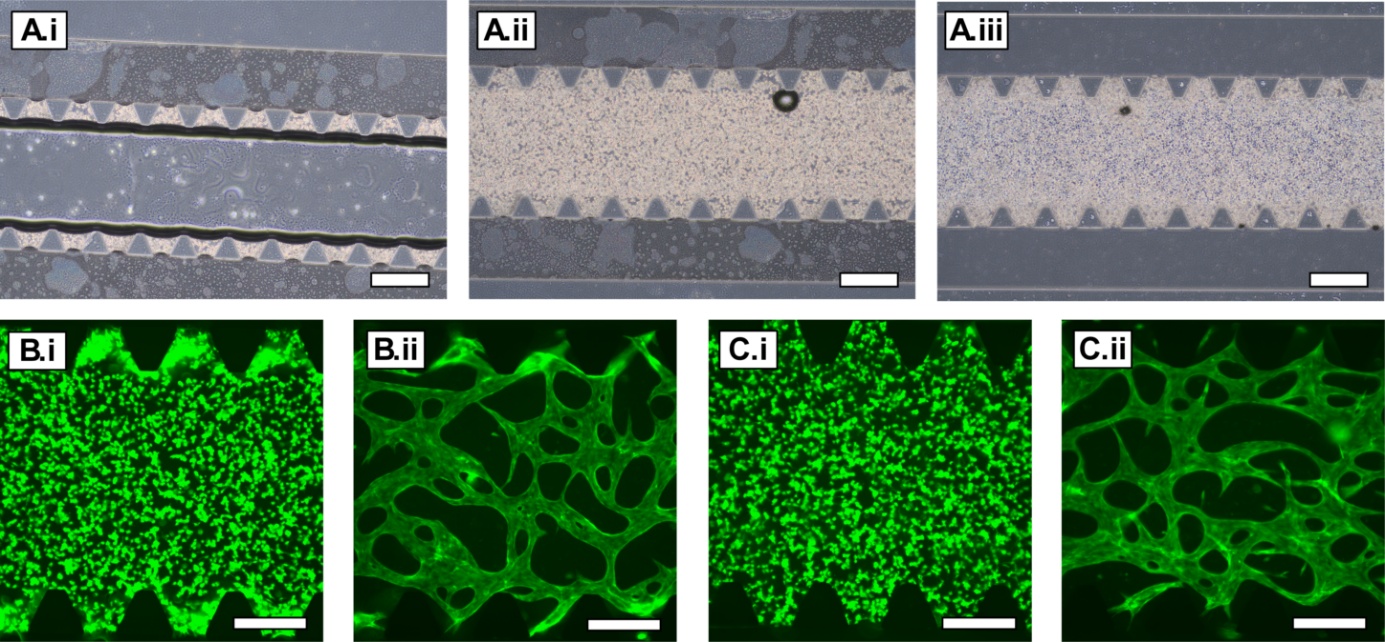


**Figure S16. An alternative seeding strategy improved inter-pillar vessels but does not support network opening.** **A.** A 100% suspension of 13 × 10^6^ GFP-hAECs/mL in hydrogel precursor is loaded into the culture compartment and immediately aspirated leaving cell suspension between pillars (i). The remainder of the culture compartment is then filled with a 1:1 GFP-hAEC:hAD-MSC hydrogel precursor suspension (ii) and gelled for 15 minutes and then channels filled with complete vascular cell media (iii, scale bars = 500 µm). **B.** Green fluorescence images of the alternate seeding strategy at days 0 (i) and 7 (ii, scale bar = 300 µm). **C.** Green fluorescence images of the typical seeding strategy at days 0 (i) and 7 (ii, scale bar = 300 µm).


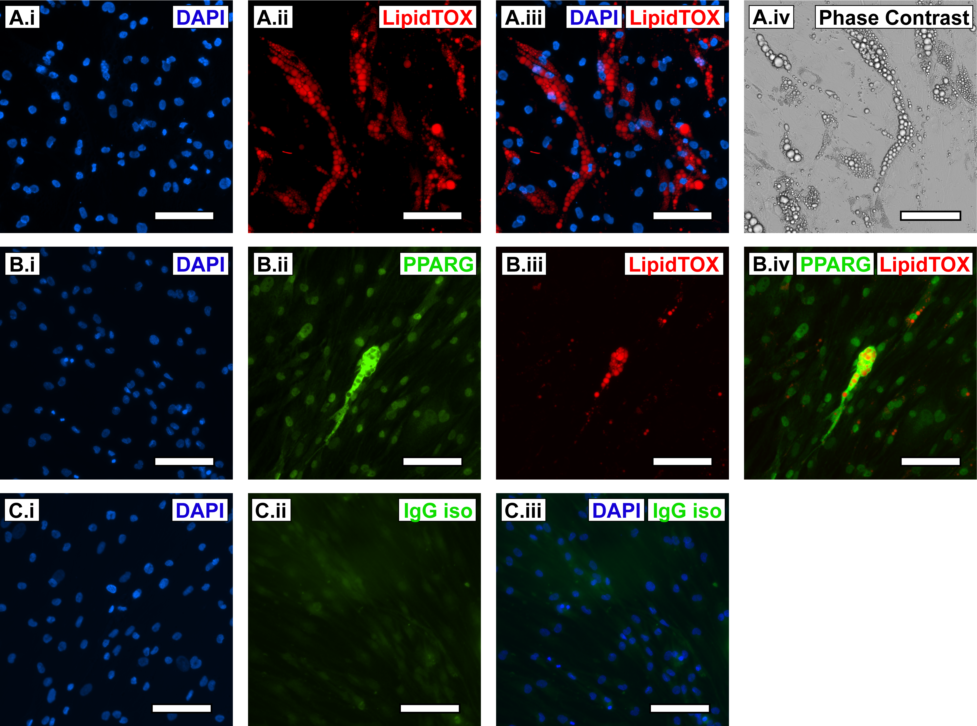
**Figure S17. hAD-MSCs exhibit a characteristic adipocyte phenotype after 17 days of 2D differentiation culture. A.** Lipid-sensitive fluorescent LipidTOX stains lipid droplets, panels depict DAPI nuclear counterstain (i), LipidTOX (ii), DAPI-LipidTOX merge (iii) and phase contrast. **B.** Immunocytochemical detection for peroxisome proliferator-activated receptor gamma (PPARG), panels depict DAPI nuclear counterstain (i), PPARG (ii), LipidTOX (iii) and PPARG-LipidTOX merge (iv). **C.** Rabbit IgG isotype control, panels depict DAPI nuclear counter stain (i), IgG isotype control antibody (ii) and DAPI-IgG isotype control antibody merge (iii). Scale bars = 100 µm.

**Supplemental Material for Growth Factor Distribution Modelling.**

Simplified code used to generate growth factor distribution models and plots like those found **figure 3**. Code has been simplified to include a single culture media change where vascular growth factor IGF-1 is added from left-hand top and bottom media channels and stromal growth factor EGF is added from right-hand top and bottom channels. The simulations create a 3D matrix of each growth factor, representing its distribution across the hydrogel culture compartment (*xy*) and time. The 3D matrices are then plotted in three ways similar as represented in **figure 3.C**, a 3D surface plot of maximal growth factor concentrations for each *xy* location in culture, a 2D line plot of average relative growth factor concentration over the length of the culture compartment over time, and a 2D heatmap of maximal relative growth factor concentrations. The second two plots have superimposed lines relating to the position where the half-maximal effective concentration of each growth factor is reached across the culture compartment.

Code can be run in Python, dependent on numpy and matplotlib.pyplot packages.

import numpy as np

import matplotlib.pyplot as plt

## LAPLACIAN function

def laplacian(Z):

Ztop = Z[0:-2, 1:-1]

Zleft = Z[1:-1, 0:-2]

Zbottom = Z[2:, 1:-1]

Zright = Z[1:-1, 2:]

Zcenter = Z[1:-1, 1:-1]

Lap = (Ztop + Zleft + Zbottom + Zright - 4 * Zcenter)

return Lap

## PARAMETERS #need dT/dX^2 to be less than 0.002

X = 1.4*10**3 #um (1 mm total) 1.4 mm wide

sizeX = 14 #1mm time steps

Y = 7.9*10**3 #um (1 mm total) 7.9 mm long

sizeY = 79 #1mm time steps

dX = ((X/sizeX) + (Y/sizeY))/2

T = 48*60*60 #s (2 days)

sizeT = 20000 #discretisation

dT = T / sizeT #8.64 second time steps

# DEX - LIMITING FACTOR FOR ADIPOGENESIS

DEX_MW = 392 #g/mol

DEX_D = 10.8 #um2/s

DEX_T50 = 4.5 * 60 * 60 #s#h

DEX_EC50 = 4.8 * 10**-3 * DEX_MW #ng/mL

DEX_Init = 1 * DEX_MW #ng/mL

# IGF-1 - LIMITING FACTOR FOR VASCULOGENESIS

IGF1_MW = 7600 #g/mol

IGF1_D = 159 #um2/s

IGF1_T50 = 0.4 * 60 * 60 #s

IGF1_EC50 = 5 #ng/mL

IGF1_Init = 15 #ng/mL

# SIMULATION FOR ADIPOGEN ON RIGHT

# INITIAL CONDITIONS

GFdiff = DEX_D

GFlife = DEX_T50

GFinit = DEX_Init

GFEC50 = DEX_EC50

GFprod = 0

GFcons = 0

rhoCell = 0

GF = np.zeros((sizeX, sizeY, sizeT))

GF[:,:,0] = np.zeros((sizeX,sizeY)) #first time step

GF[0,int(0.68*sizeY):(sizeY),:] = np.ones((int(0.34*sizeY),sizeT))*GFinit #bottom right condition

GF[sizeX-1,int(0.68*sizeY):(sizeY),:] = np.ones((int(0.34*sizeY),sizeT))*GFinit #top right condition

# SIMULATION

for t in range(0,sizeT-1):

dGF = laplacian(GF[:,:,t])

GFc = GF[1:-1, 1:-1, t] #last iteration

GFtemp = GFc + GFdiff*dT/np.power(dX,2)*dGF + (np.log(0.5)/GFlife)*GFc

GFtemp = np.row_stack((GFtemp[0,:], GFtemp, GFtemp[-1, :]))

GFtemp = np.column_stack((GFtemp[:, 0], GFtemp, GFtemp[:, -1]))

GFtemp[0,int(0.68*sizeY):(sizeY)] = np.ones(int(0.34*sizeY))*(GFinit*np.exp(t*np.log(0.5)/GFlife)) #bottom right condition

GFtemp[sizeX-1,int(0.68*sizeY):(sizeY)] = np.ones(int(0.34*sizeY))*(GFinit*np.exp(t*np.log(0.5)/GFlife)) #top right condition

GF[:,:,t+1] = GFtemp

ADIPORIGHT = GF

# SIMULATION FOR ANGIOGEN ON LEFT

GFdiff = IGF1_D

GFlife = IGF1_T50

GFinit = IGF1_Init

GFEC50 = IGF1_EC50

GFprod = 0

GFcons = 0

rhoCell = 0

GF = np.zeros((sizeX, sizeY, sizeT))

GF[:,:,0] = np.zeros((sizeX,sizeY)) #first time step

GF[0,0:int(0.34*sizeY),:] = np.ones((int(0.34*sizeY),sizeT))*GFinit #bottom left condition

GF[sizeX-1,0:int(0.34*sizeY),:] = np.ones((int(0.34*sizeY),sizeT))*GFinit #top left condition

for t in range(0,sizeT-1):

dGF = laplacian(GF[:,:,t])

GFc = GF[1:-1, 1:-1, t] #last iteration

GFtemp = GFc + GFdiff*dT/np.power(dX,2)*dGF + (np.log(0.5)/GFlife)*GFc

GFtemp = np.row_stack((GFtemp[0,:], GFtemp, GFtemp[-1, :]))

GFtemp = np.column_stack((GFtemp[:, 0], GFtemp, GFtemp[:, -1]))

GFtemp[0,0:int(0.34*sizeY)] = np.ones(int(0.34*sizeY))*(GFinit*np.exp(t*np.log(0.5)/GFlife)) #bottom left condition - 1 ng/mL is 10^-12 ng/um3

GFtemp[sizeX-1,0:int(0.34*sizeY)] = np.ones(int(0.34*sizeY))*(GFinit*np.exp(t*np.log(0.5)/GFlife)) #top left condition - 1 ng/mL is 10^-12 ng/um3

GF[:,:,t+1] = GFtemp

ANGIOLEFT = GF

# PLOTTING 3D SURFACE OF CONCENTRATIONS (i.e. Fig. 5.B.i)

Oranges = np.nanmax(ADIPORIGHT,axis=2)

np.nanargmin(ADIPORIGHT,axis=2)

Oranges = np.fliplr(Oranges)

plt.figure()

Ym = np.arange(0, X, 100)

Xm = np.arange(0, Y, 100)

Xm, Ym = np.meshgrid(Xm, Ym)

fig, ax = plt.subplots(subplot_kw={"projection": "3d"})

surf = ax.plot_surface(Xm, Ym, Oranges, cmap='Oranges', linewidth=0, antialiased=False)

fig = plt.title('Concentration (ng/mL)')

plt.show()

# PLOTTING 2D TIME PROFILES (i.e. Fig. 5.B.ii)

plt.figure()

GF2D = ADIPORIGHT[int(X/(dX*2)),:,:]

for t in range(1,int(T//dT-1),int((T//dT-1)/(sizeX-1))):

plt.plot(np.linspace(0,Y-dX,num=sizeY)/1000,GF2D[:,t]/np.max(GF2D),color=(((sizeT-t)/sizeT)*0.941, ((sizeT-t)/sizeT)*0.5, ((sizeT-t)/sizeT)*0.125))

TFarray = np.amax(GF2D>DEX_EC50,axis=1).astype(float)

plt.axvline(x = np.argmin(TFarray[:-1]-TFarray[1:])/10, color = (0.941, 0.5, 0.125), linestyle = '--')

GF2D = ANGIOLEFT[int(X/(dX*2)),:,:]

for t in range(1,int(T//dT-1),int((T//dT-1)/(sizeX-1))):

plt.plot(np.linspace(0,Y-dX,num=sizeY)/1000,GF2D[:,t]/np.max(GF2D),color=(((sizeT-t)/sizeT)*0.941, ((sizeT-t)/sizeT)*0.125, ((sizeT-t)/sizeT)*0.125))

TFarray = np.flip(np.amax(GF2D>IGF1_EC50,axis=1).astype(float))

plt.axvline(x = 7.8-np.argmin(TFarray[:-1]-TFarray[1:])/10, color = (0.941, 0.125, 0.125), linestyle = '--')

plt.title('Distribution over Time')

plt.ylabel('Relative Concentration at Centreline')

plt.xlabel('Length (mm)')

plt.show()

# PLOTTING 2D SURFACE OF COUNTERCURRENT CONCENTRATIONS (i.e. Fig. 5.B.iii)

blur_start1 = 26

blur_end1 = 40

blur_start2 = 45

blur_end2 = 55

alphalr = np.ones(np.shape(ANGIOLEFT[:,:,0]))

alphalr[:,blur_start1:] = np.concatenate((np.linspace(1, 0, blur_end1-blur_start1), np.zeros(np.shape(ANGIOLEFT)[1]-blur_end1)))

alpharl = np.ones(np.shape(ADIPORIGHT[:,:,0]))

alpharl[:,:-blur_start2] = np.concatenate((np.zeros(np.shape(ADIPORIGHT)[1]-blur_end2),np.flip(np.linspace(1, 0, blur_end2-blur_start2))))

plt.figure()

Greens = np.nanmax(ANGIOLEFT,axis=2) # plots maximum distribution of factors – can substitute for a selected time interval by using ANGIOLEFT[:,:,time]

Oranges = np.nanmax(ADIPORIGHT,axis=2)

plt.imshow(Greens, cmap= 'Reds', origin='lower', interpolation='none', vmin=0, alpha=alphalr, vmax=IGF1_Init)

plt.imshow(Oranges, cmap= 'Oranges', origin='lower', interpolation='none', vmin=0, alpha=alpharl, vmax=DEX_Init)

TFarray = np.amax(ANGIOLEFT,axis=2)>IGF1_EC50 #plotting intersections with EC50 concentrations

Line = (TFarray[:,0:-1].astype(int) - TFarray[:,1:].astype(int)).astype(float)

Line[Line == 0] = np.nan

plt.imshow(Line, cmap= 'Reds', vmin=0, vmax=1)

TFarray = np.fliplr(np.amax(ADIPORIGHT,axis=2)>DEX_EC50)

Line = np.fliplr((TFarray[:,0:-1].astype(int) - TFarray[:,1:].astype(int)).astype(float))

Line[Line == 0] = np.nan

plt.imshow(Line, cmap= 'Oranges', vmin=0, vmax=1)

plt.ylabel("Max Dist.")

ax.yaxis.set_ticks([10, 0])

ax.yaxis.set_ticklabels(['1', '0'])

ax.xaxis.set_ticks([0, 10, 20, 30, 40, 50, 60, 70, 79])

ax.xaxis.set_ticklabels(['0', '1', '2', '3', '4', '5', '6', '7', '7.9'])

plt.tight_layout()

plt.show()

**
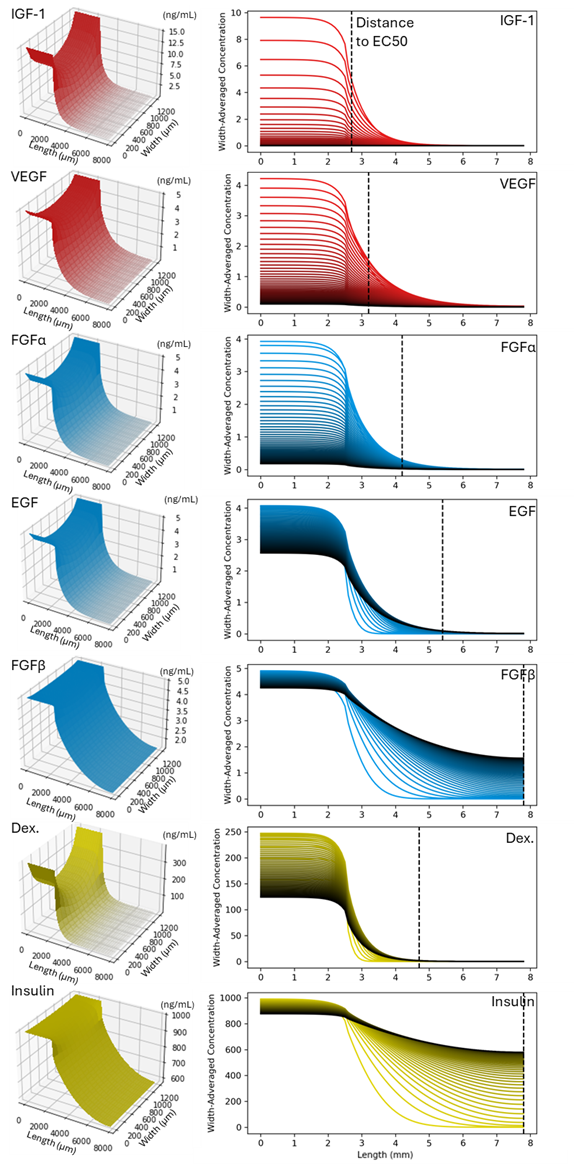
**

**Figure S18. Simulated gradients of stromal maintenance, vasculogenic and adipogenic growth factors.** The vascular (Vasc, red), stromal (MSC, blue), adipogenic (Adipo, yellow) growth factors IGF-1, VEGF, FGF-α, EGF, FGF-β, DEX and insulin. The maximum simulated concentration (in ng/mL) of these factors for each mm^2^ of culture compartment area (13 × 79 mm) over 2 days (i). Growth factor concentrations across the culture compartment length at mid-width, relative to supplemented concentrations, where line colour turns darker for each hour of culture up to 2 days (ii). IGF-1: insulin-like growth factor 1, VEGF: vascular endothelial growth factor, FGF-α: fibroblast growth factor acidic, EGF: epidermal growth factor, FGF-β: fibroblast growth factor basic, DEX: dexamethasone.


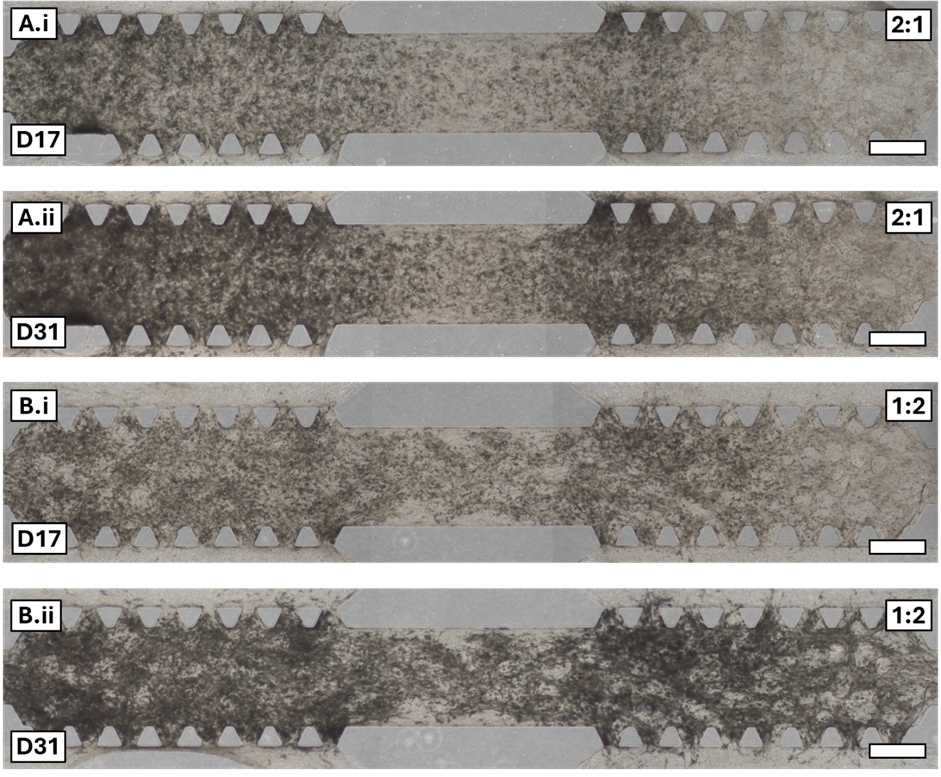


**Figure S19. GFP-hAEC) and hAD-MSC gradient co-culture supports the co-formation of microvascular networks and adipogenic differentiation. A.** Colour brightfield microscopy images of 2:1 seeding ratio microfluidic device cultures after 17 (i) and 31-days (ii, scale bars = 500 µm). **B.** Colour brightfield microscopy images of 1:2 seeding ratio microfluidic device cultures after 17 (i) and 31-days (ii, scale bars = 500 µm).


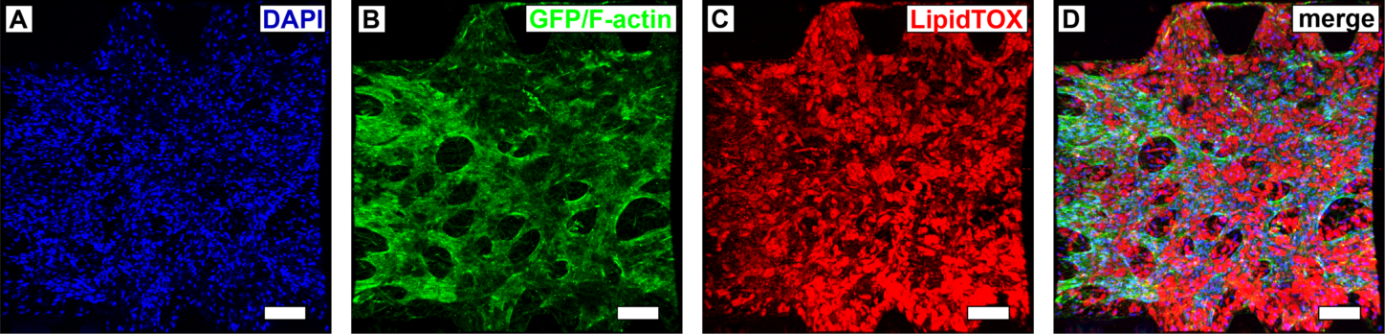


**Figure S20. GFP-hAEC and hAD-MSC gradient co-culture supports the co-formation of microvascular networks and adipocyte differentiation.** Low magnification images of 1:1 seeding ratio cultures at day 31. **A.** DAPI nuclear counterstain. **B.** F-actin (green) and GFP (green). **C.** LipidTOX (red). **D.** A merge of the three channels (scale bars = 150 µm).


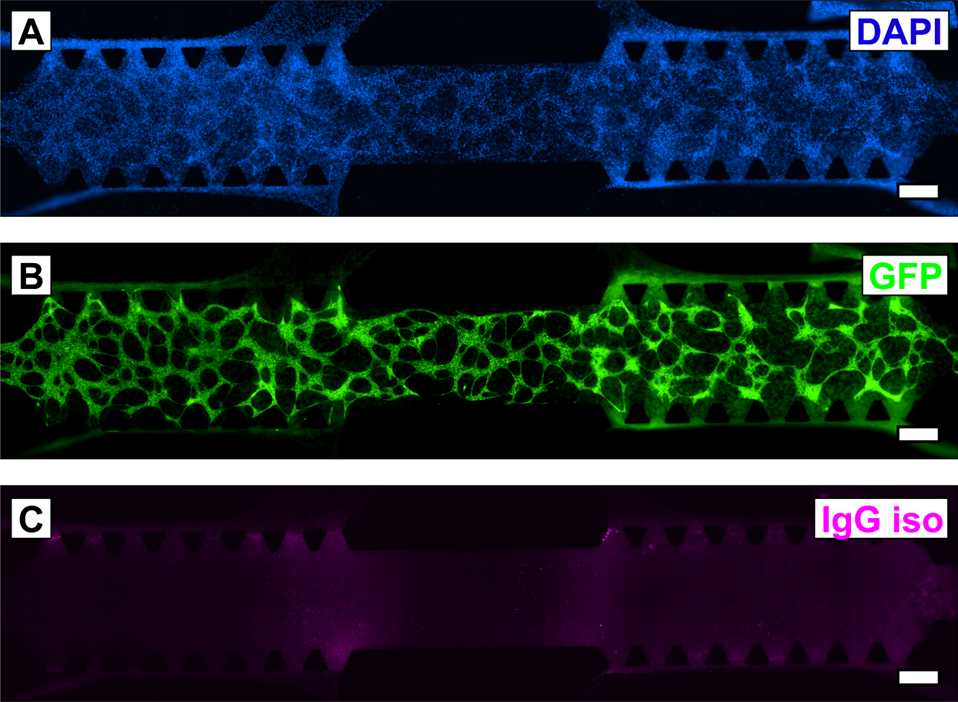


**Figure S21. Immunocytochemical detection of 1:1 co-cultures at day 17 for IgG isotype control antibody.** Fluorescence mages depicting the nuclear counter stain DAPI (*A*), GFP (*B*) and rabbit IgG isotype control (*C*, scale bars = 500 µm).


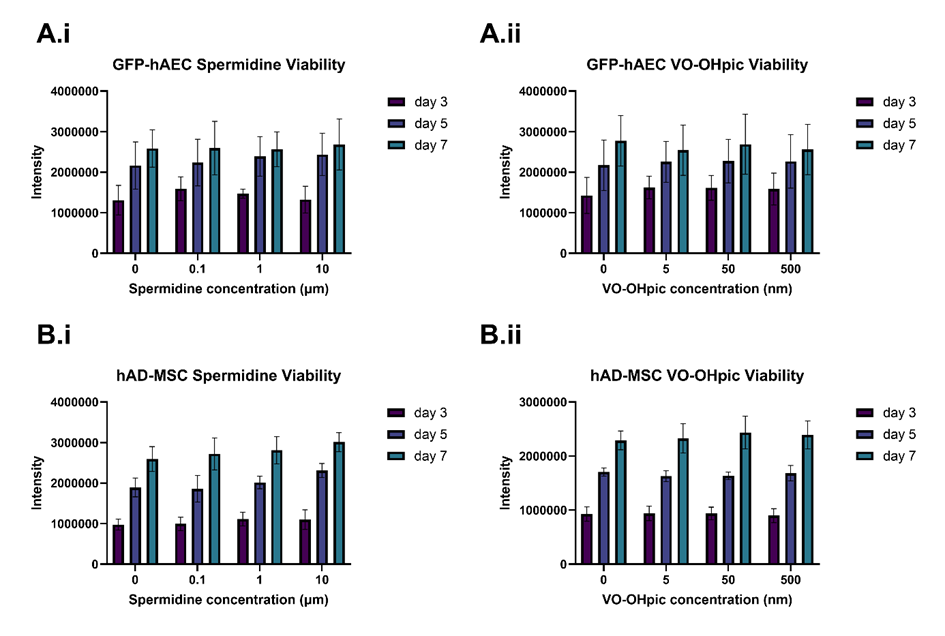


**Figure S22. Both spermidine and hydroxyl(oxo)vanadium 3-hydroxypiridine-2-carboxylic acid (VO-OHpic) exhibit no significant influence on GFP-hAEC or hAD-MSC mono-cultures. A.** GFP-hAECs cultured for 3, 5 and 7-days in media supplemented with 0–10 µM spermidine (i) and 0–500 nM VO-OHpic (ii) (n = 3 biological replicas, mean ± standard deviation). Results were compared via an ordinary two-way ANOVA followed by a Tukey multiple comparisons test (α = 0.05). **B.** hAD-MSCs cultured for 3, 5 and 7-days in media supplemented with 0–10 µM spermidine (i) and 0–500 nM VO-OHpic (ii) (n = 3 biological replicas, mean ± standard deviation). Results were compared via an ordinary two-way ANOVA followed by a Tukey multiple comparisons test.


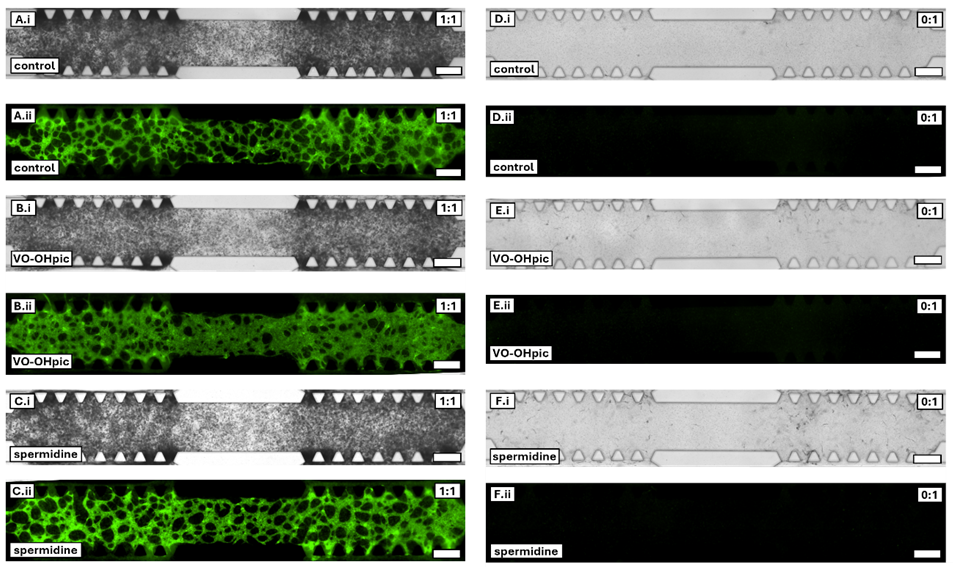


**Figure S23. Inducers of lipolysis do not inhibit the formation of lipids in GFP-hAEC and hAD-MSC gradient co-cultures.** Monochromatic brightfield (i) and green fluorescence (ii) images of day 31 1:1 seeding ratio cultures without lipolytic supplements (**A**) with 50 nM VO-OHpic (**B**) and 1 µM spermidine (**C**, scale bars = 500 µm). Monochromatic brightfield (i) and green fluorescence (ii) images of day 31 0:1 seeding ratio cultures without lipolytic supplements (**D**) with 50 nM VO-OHpic (**E**) and 1 µM spermidine (**F**, scale bars = 500 µm).
